# Longitudinal evaluation of neuroinflammation and oxidative stress in a mouse model of Alzheimer disease using positron emission tomography

**DOI:** 10.1186/s13195-022-01016-5

**Published:** 2022-06-09

**Authors:** Luka Rejc, Vanessa Gómez-Vallejo, Ana Joya, Gemma Arsequell, Ander Egimendia, Pilar Castellnou, Xabier Ríos-Anglada, Unai Cossío, Zuriñe Baz, Leyre Iglesias, Estibaliz Capetillo-Zarate, Pedro Ramos-Cabrer, Abraham Martin, Jordi Llop

**Affiliations:** 1 CIC biomaGUNE, Basque Research and Technology Alliance (BRTA), Paseo Miramon 182, 20014 San Sebastian, Spain; 2 Laboratory of Neuroimaging and biomarkers of inflammation, UPV/EHU, Sede building B. Sarriena, Achucarro Basque Center for Neuroscience, 48940 Science ParkLeioa, Spain; 3 Institut de Química Avançada de Catalunya (IQAC), Spanish National Research Council (IQAC-CSIC), 08034 Barcelona, Spain; 4 Faculty of Medicine and Nursery, University of the Basque Country UPV/EHU, Achucarro Basque Center for Neuroscience and CIBERNED, Barrio Sarriena S/N, 48940 Leioa, Spain; 5 Basque Foundation for Science, IKERBASQUE, 48009 Bilbao, Spain

**Keywords:** TSPO, Oxidative stress, Positron emission tomography, Alzheimer disease

## Abstract

**Background:**

Validation of new biomarkers of Alzheimer disease (AD) is crucial for the successful development and implementation of treatment strategies. Additional to traditional AT(N) biomarkers, neuroinflammation biomarkers, such as translocator protein (TSPO) and cystine/glutamine antiporter system (x_c_^-^), could be considered when assessing AD progression. Herein, we report the longitudinal investigation of [^18^F]DPA-714 and [^18^F]FSPG for their ability to detect TSPO and x_c_^-^ biomarkers, respectively, in the 5xFAD mouse model for AD.

**Methods:**

Expression of TSPO and x_c_^-^ system was assessed longitudinally (2–12 months of age) on 5xFAD mice and their respective controls by positron emission tomography (PET) imaging using radioligands [^18^F]DPA-714 and [^18^F]FSPG. In parallel, in the same mice, amyloid-β plaque deposition was assessed with the amyloid PET radiotracer [^18^F]florbetaben. In vivo findings were correlated to ex vivo immunofluorescence staining of TSPO and x_c_^-^ in microglia/macrophages and astrocytes on brain slices. Physiological changes of the brain tissue were assessed by magnetic resonance imaging (MRI) in 12-month-old mice.

**Results:**

PET studies showed a significant increase in the uptake of [^18^F]DPA-714 and [^18^F]FSPG in the cortex, hippocampus, and thalamus in 5xFAD but not in WT mice over time. The results correlate with Aβ plaque deposition. Ex vivo staining confirmed higher TSPO overexpression in both, microglia/macrophages and astrocytes, and overexpression of x_c_^-^ in non-glial cells of 5xFAD mice. Additionally, the results show that Aβ plaques were surrounded by microglia/macrophages overexpressing TSPO. MRI studies showed significant tissue shrinkage and microstructural alterations in 5xFAD mice compared to controls.

**Conclusions:**

TSPO and x_c_^-^ overexpression can be assessed by [^18^F]DPA-714 and [^18^F]FSPG, respectively, and correlate with the level of Aβ plaque deposition obtained with a PET amyloid tracer. These results position the two tracers as promising imaging tools for the evaluation of disease progression.

**Graphical abstract:**

Longitudinal in vivo study in the 5xFAD mouse model shows that TSPO and oxidative stress assessment through [^18^F]DPA-714 and [^18^F]FSPG-PET imaging, respectively, could serve as a potential tool for the evaluation of Alzheimer disease progression.

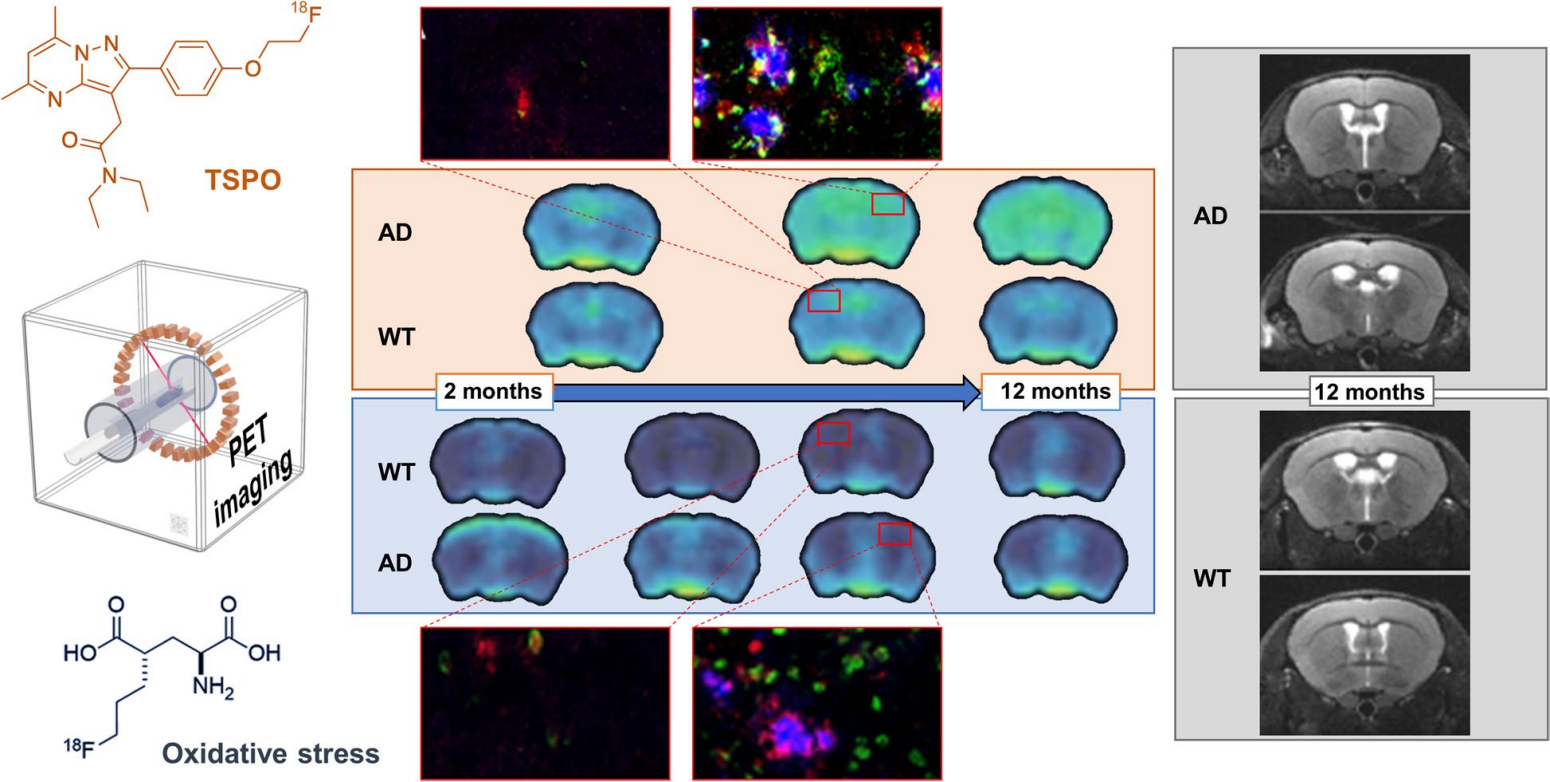

**Supplementary Information:**

The online version contains supplementary material available at 10.1186/s13195-022-01016-5.

## Background

Alzheimer disease (AD) is the most common cause of dementia and affects over 13 million people worldwide. This number is expected to increase to more than 100 million by 2050 [1]. There is no effective treatment for AD available on the market and, more worryingly, no reliable option for the early diagnosis of the disease. The recent approval of aducanumab by the FDA (Aduhelm) [2] has been an important milestone in disease intervention. However, the treatment was not as effective as it was initially expected. One of the responsible factors for this was hypothesized to be the disease stage of the patients enrolled in the clinical trials [3], and a review of the study suggests a lack of correlation between surrogate imaging biomarkers and clinical outcome [4]. These facts put a spotlight on both early diagnosis and the development of surrogate markers capable of evaluating and predicting disease progression.

Pathophysiologically, AD is characterized by the accumulation of amyloid-β (Aβ) aggregates in various conformations [5], the appearance of filamentous intraneuronal inclusions made of hyperphosphorylated Tau protein (p-Tau) [6], and synaptic dysfunction [7]. Some of these processes begin decades before the onset of clinical symptoms, which is why the most recent AT(N) scheme, published by the National Institute on Aging and Alzheimer’s Association, proposed establishing a definition of AD based on the assessment of disease biomarkers, namely Aβ plaque (A), fibrillary tau (T), and neurodegeneration or neuronal damage (N), and not on clinical symptoms [8]. Apart from characteristic pathology, AD patients also exhibit altered glucose metabolism [9], neuroinflammation [10], and oxidative stress [11]. In fact, oxidative stress and neuroinflammation were suggested to play the key role in the pathophysiology of neurodegeneration and promoted cognitive decline of dementia patients [12]. Furthermore, studies show that microglia and astroglia cells were activated in 5xFAD mice at the onset of Aβ plaque formation [13]. The role of oxidative stress and neuroinflammation in the onset of AD and its progression, and their association with Aβ plaques [14, 15], places them as a potential source of (imaging) biomarkers for AD.

In vivo, neuroinflammation and oxidative stress can be detected indirectly by assessing overexpression of the translocator protein (18 kDa) (TSPO) and the cystine/glutamate antiporter (x_c_^-^) system, respectively [16, 17]. A large pool of literature data indicates that TSPO is a promising target to monitor glial cell and infiltrated macrophage activation during the inflammation process [18–20]. Additionally, autoradiography using [^3^H]PBR28 (TSPO-specific radioligand) showed an increase in specific binding in 5xFAD compared to WT mice, confirming a strong relationship between neuroinflammation and upregulation of TSPO [21, 22]. On the other hand, overexpression of the x_c_^-^ transporter leads to increased levels of antioxidant glutathione through an increased transport of cystine [23]. This makes this antiporter system one of the possible targets for assessment of oxidative stress by PET imaging, using radioactively labeled x_c_^-^ substrates, such as glutamate analogue, (4S)-4-(3-[^18^F]fluoropropyl)-L-glutamate ([^18^F]FSPG) [24]. Although there has been some dispute over the ability of [^18^F]FSPG to cross intact blood-brain barrier (BBB), some studies indicate that limited transport into the brain could be possible [25, 26], and it has been successfully applied to investigate oxidative stress in cancer [27] and different neurological disorders, such as cerebral ischemia [20] and multiple sclerosis [28].

In this work, we report the longitudinal evaluation of [^18^F]DPA-714 and [^18^F]FSPG to assess the levels of neuroinflammation and oxidative stress, respectively, in the 5xFAD mouse model for AD by PET imaging in vivo. The results have been correlated to Aβ plaque burden, as determined by PET imaging using the validated radiotracer [^18^F]florbetaben and previously reported by our research group [29].

## Materials and methods

### General aspects

Animal handling was conducted in accordance with the European Council Directive 2010/63/UE. All experimental procedures were approved by the Ethical Committee at CIC biomaGUNE and local authorities (PRO-AE-SS-095).

Amyloid beta overexpressing female transgenic hemizygous 5xFAD mice (B6SJL-Tg(APPSwFlLon,PSEN1*M146L*L286V)6799Vas/Mmjax) and control female wild type (WT) C57BL/6J × SJL/J F1 mice were obtained from The Jackson Laboratory (Bar Harbor, ME, USA) in two batches (batch #1: *n*(AD) = 10, *n*(WT) = 8; batch #2: *n*(AD) = 13, *n*(WT) = 10) at the age of 10 weeks. The same animals were used in another longitudinal study, recently reported by our research group [29], where butyrylcholinesterase inhibitor (named as [^11^C]**4** in the original work) and [^18^F]florbetaben were used as PET tracers. Females were selected because (i) the prevalence of AD is higher among women than among men with a 2:1 women/men ratio and (ii) the disease in this strain is more aggressive in females than in males [30].

### Positron emission tomography (PET) imaging studies

**Fig. 1:**
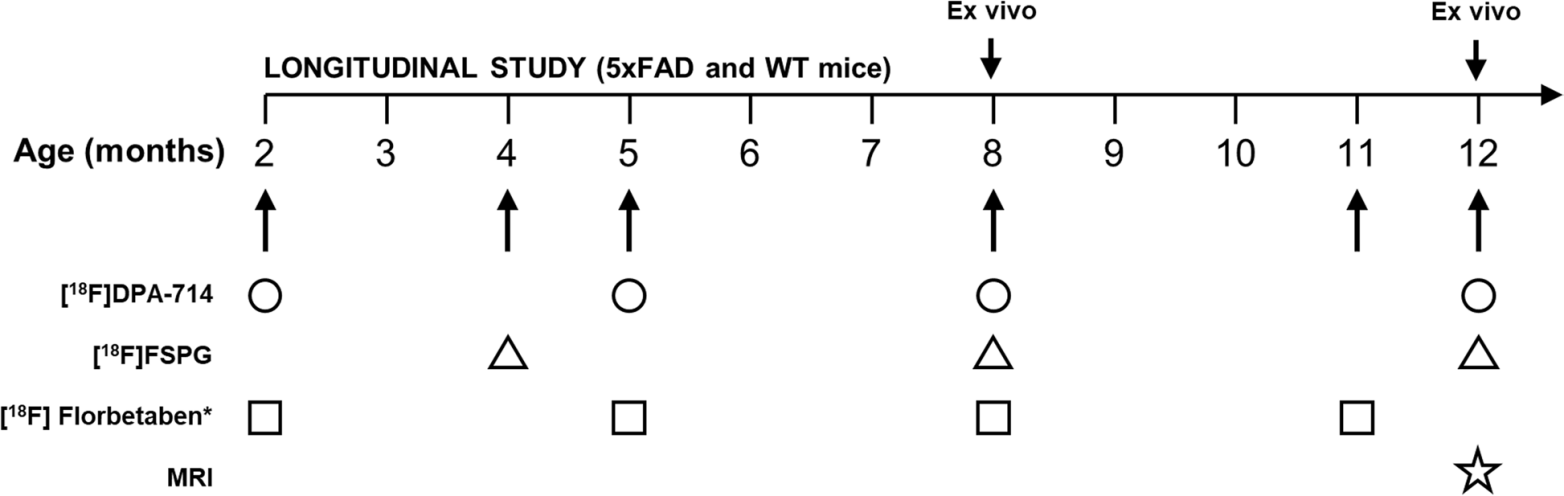
Study design: 5xFAD and WT mice were injected with [^18^F]DPA-714, [^18^F]FSPG, and [^18^F]florbetaben, at different times. MRI studies were carried out at 12 months of age. ^*^Previously reported in [29]

For PET imaging studies included in the current work, mice were injected intravenously under anesthesia with [^18^F]DPA-714 [31] at 4, 8, and 12 months of age and [^18^F]FSPG [20] at 2, 5, 8, and 12 months of age (see ESI for more details on radiotracer preparation and experimental details; see Fig. 1 and Table S1 for imaging studies performed on each animal, including those reported in our previous work; see Table S2 for the amount of radioactivity administered to each animal to perform [^18^F]DPA-714 and [^18^F]FSPG PET studies).

In all cases, imaging was performed during the light phase of the light–dark cycle. Dynamic PET-CT images (eXplore Vista-CT; GE Healthcare, WI, USA) were acquired in one bed position, with the brain centered in the middle of the field of view (FOV) to acquire the dynamic distribution in the brain (time of scan = 59.4 min). After reconstruction (filtered back projection (FBP) applying random, scatter, and attenuation corrections) and co-registration (M. Mirrione-T2 MRI template), uptake of radioactivity was determined in the cortex (CTX), the hippocampus (HIP), the thalamus (THA) (these regions showing increased beta-amyloid plaque burden at age > 3 months in this animal model [32]), the cerebellum (CB), and the whole brain (WB) using the π-MOD image analysis software (π-MOD Technologies Ltd, Zurich, Switzerland). In case of [^18^F]DPA-714, the CB was selected as the reference region [33], and images were analyzed using Regional Logan Plot analysis to determine the distribution volume ratio (DVR). In case of [^18^F]FSPG, uptake values standardized to the animal weight (SUV) were determined by averaging the signal in the last 20 min of the dynamic PET scan.

### Magnetic resonance imaging (MRI) studies

MR imaging was performed as a single imaging session at the end of the longitudinal PET studies at 11.7 Tesla on a Bruker Biospec 117/16 USR scanner (Bruker Biospin, Ettlingen, Germany). The imaging protocol included the acquisition of 3 orthogonal T_1_W sets of images, a set of high-resolution T_2_-weighted images, and a set of diffusion tensor imaging (DTI). Maps of fractional anisotropy (FA), mean diffusivity (MD), radial diffusivity (RD), and axial diffusivity (RD) were calculated using Dipy library for python (see the supplementary information for detailed information on MRI image acquisition and processing).

### Ex vivo studies

To corroborate PET data, representative animals of each group were sacrificed at 8 months of age. For these animals and those sacrificed at 12 months of age, the brain was harvested for ex vivo immunofluorescence and Thioflavin staining. Different brain sections containing CTX, HIP, THA, and CB were stained separately for Iba1, GFAP, TSPO, and xCT (light subunit of the xCT-4F2hc heterodimer known as system x_c_^-^ [20]) to evaluate the cellular expression of both TSPO and xCT in microglia/macrophages and astrocytes. Additionally, Thioflavin S staining was performed on tissue sections. Images were acquired with the Pannoramic MIDI II automated digital slide scanner (3DHistech Ltd., Hungary).

### Statistical analysis

PET results were analyzed using a two-way analysis of variance ANOVA. Differences between groups (5xFAD vs WT) at each time point and differences between time points within each group were determined using Sidak’s multiple comparisons test. For MRI results, the statistical significance of the difference between groups was determined by a two-way ANOVA, followed by an unpaired *t*-test. Differences were concluded significant for *P* values < 0. 05: *P* < 0.05, *; *P* < 0.01, **; *P* < 0.001, ***; and *P* < 0.0001, ****. Statistical tests were performed in GraphPad Prism 7.03 (GraphPad Software, CA, USA).

## Results

### PET-[^18^F]DPA-714

**Fig. 2:**
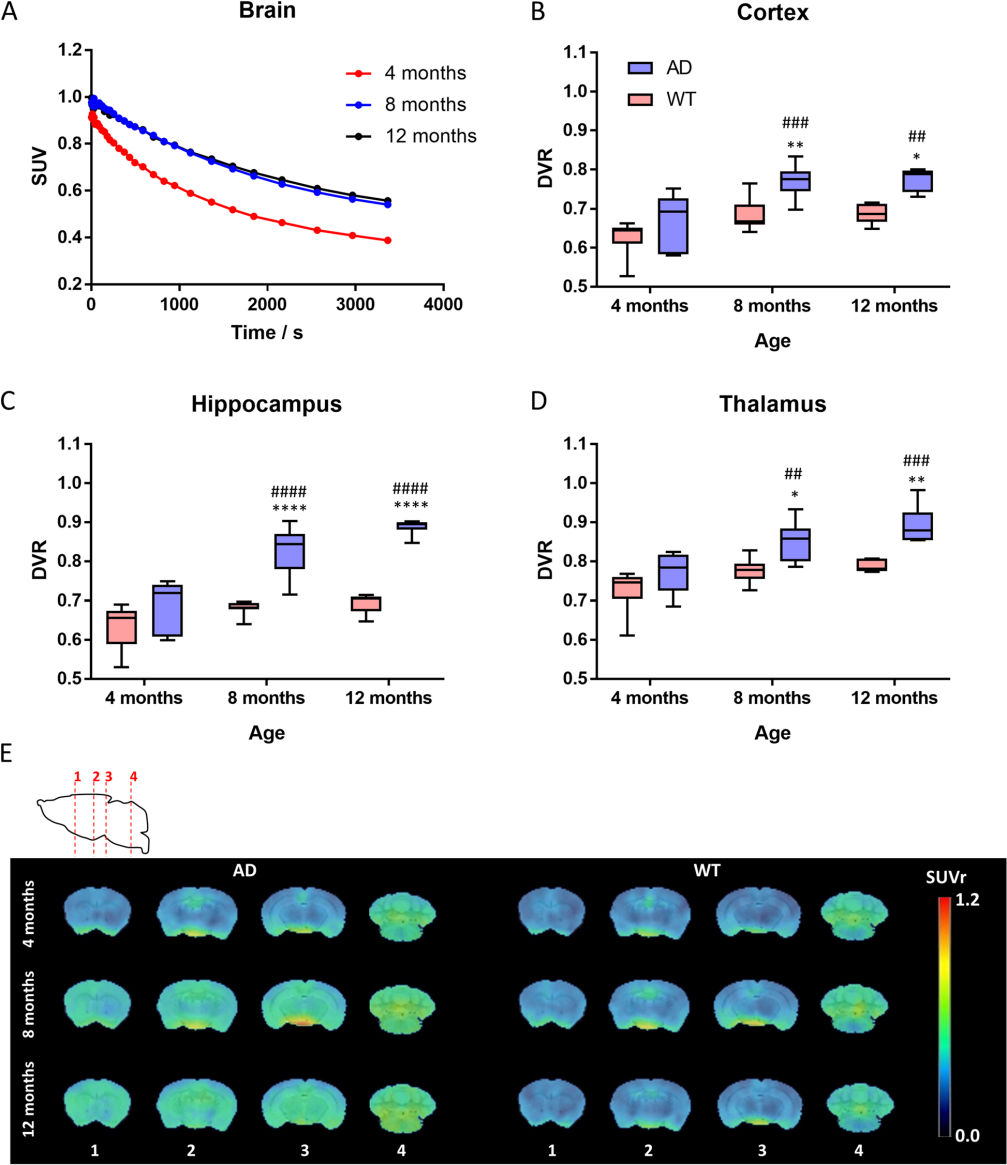
**A** Time-activity curves in the whole brain, expressed in standard uptake value (SUV) after administration of [^18^F]DPA-714 to 5xFAD mice at the age of 4, 8, and 12 months. **B**–**D** Box plots representing distribution volume ratios (DVRs) in the cortex, hippocampus, and thalamus, obtained by Regional Logan Plot analysis (the cerebellum as the reference region). “*” depicts the difference between groups at each time point and “#” depicts the difference within group with respect to the starting time point (4 months). Probability values are depicted as * (***P*** < 0.05), ** (***P*** < 0.01), *** (***P*** < 0.001), and **** (***P*** < 0.0001). **E** PET images (representative axial slices) obtained in AD and WT animals after intravenous administration of [^18^F]DPA-714 at different ages. Images have been generated by dividing, voxel-by-voxel, averaged images (last three frames) by the average value of the concentration of radioactivity in the cerebellum (SUVr), considered as the reference region

At the age of 4 months, WB time-activity curves (TACs) of AD animals showed a rapid increase in [^18^F]DPA-714 uptake after injection, followed by a progressive decrease that persisted to the end of the PET study (Fig. 2A).

A similar profile was obtained for animals aged 8 and 12 months. However, in these cases, the progressive decrease after the initial peak was slower, suggesting a higher retention of the tracer in the brain. For WT animals (Fig. S1A), similar profiles were obtained for 4-, 8-, and 12-month-aged animals, all showing progressive decrease after the initial peak. A lack of plateau within the duration of the PET imaging (Figs. 2A and S1A) compelled us to apply Regional Logan Plot analysis using CB as the reference region, to determine DVR values in CTX, HIP, and THA, for both WT and 5xFAD animals.

DVRs in CTX, HIP, and THA remained constant in WT animals and increased in 5xFAD mice over time (Fig. 2B–D), as can be visualized in averaged PET images (Fig. 2E). A closer look at the examined brain regions showed higher [^18^F]DPA-714-specific uptake in 5xFAD compared to WT mice at 4 months of age, although differences were not statistically significant. Consistently throughout these brain regions, a significant increase of DVRs was observed in 5xFAD mice at 8 months of age, resulting in 15%, 20%, and 11% higher uptake in CTX (*P* = 0.0032), HIP (*P* < 0.0001), and THA (*P* = 0.0104) of 5xFAD compared to WT animals, respectively. Comparing 4- with 12-month-old 5xFAD mice shows a significant increase of the radiotracer-specific uptake in the three brain subregions over time, namely 16% in CTX (*P* = 0.0011), 29% in HIP (*P* < 0.0001), and 16% in THA (*P* = 0.0002). At 12 months of age, the average DVRs in 5xFAD mice were higher from WT mice by 12% in CTX (*P* = 0.0119), 28% in the HIP (*P* < 0.0001), and 13% in THA (*P* = 0.0024).

### PET-[^18^F]FPSG

**Fig. 3:**
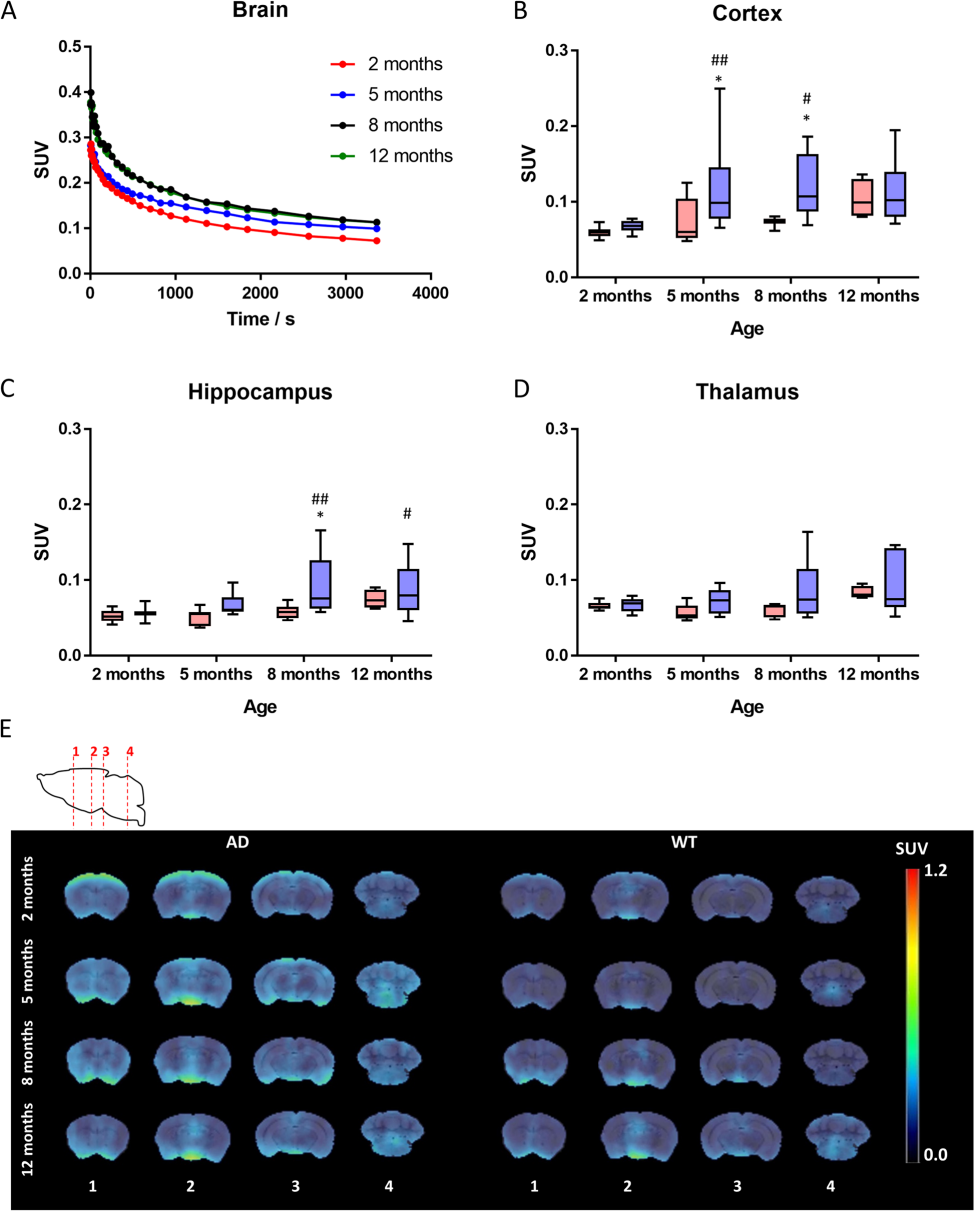
**A** Time-activity curves in the whole brain, expressed as standard uptake value (SUV) obtained after administration of [^18^F]FSPG to 5xFAD mice at the ages of 2, 5, 8, and 12 months. **B**–**D** Box plots representing SUVs in the cortex, hippocampus, and thalamus. * depicts the difference between groups at each time point and # depicts the difference within group with respect to the starting time point (2 months). Probability values are depicted as * (***P*** < 0.05) and ** (***P*** < 0.01). **E** PET images (representative axial slices) obtained in AD and WT animals after intravenous administration of [^18^F]FSPG at different ages. Images have been generated by averaging SUV images of all animals within each group and time point

Whole brain SUV curves in both AD (Fig. 3A) and WT animals (Fig. S1B) for [^18^F]FSPG showed a progressive decrease from the start of the acquisition (*ca.* 30 s after administration) suggesting that the concentration of radioactivity in the brain peaks at short times after administration. In AD animals (Fig. 3A), a longitudinal increase in the concentration of radioactivity in WB can be observed by visual comparison of TACs at 2, 5, and 8 months of age. There is no notable difference between TACs obtained for 8- and 12-month-aged animals. Contrarily, for WT animals (Fig. S1B), whole brain TACs obtained at 2, 5, and 8 months of age are very similar, while an increase in averaged values can be observed at 12 months.

The lack of appropriate reference region rendered the quantification as SUV ratios; hence, the average SUV of the last three frames of each imaging session was selected for comparison. Lower brain uptake values than for [^18^F]DPA-714 were observed. This, together with the lack of reference region and the presence of radioactivity outside of the brain, which could lead to a spill-over effect, caused a higher standard deviation of the results.

Consistent with a visual inspection, quantification of the data from longitudinal assessment of WT mice revealed no significant increase of [^18^F]FSPG uptake in CTX, HIP, and THA between 2 and 8 months of age (Fig. 3B–D). Interestingly, at the last, 12-month time point, a more abrupt increase in SUV was observed in all three brain regions. Compared to the 2-month time point, the SUV average was higher by 74% in CTX, 30% in HIP, and 21% in THA, but was not statistically significant. In contrast, 5xFAD mice exhibited an increase in [^18^F]FSPG uptake earlier. Comparing 5 months with 2-month-old 5xFAD mice, the radiotracer uptake was significantly higher by 78% in CTX (*P* = 0.0061) and not significantly higher in HIP and THA (21% and 7%, respectively). Imaging sessions at later time points showed that [^18^F]FSPG uptake in CTX remained constant and increased in HIP and THA. At 8 months of age, the average SUV increased by 62% from the 2-month time point in HIP. Similarly, 5xFAD mice exhibited an increase in THA at 8 and 12 months of age (28% and 38% increase from the 2-month time point), but compared to control mice, the difference was not statistically significant. The difference between the radiotracer uptake in WT and diseased mice was significant at 5- and 8-month time points in CTX (*P* = 0.0389 and *P* = 0.0386 at 5 and 8 months, respectively) and at 8-month time point in HIP (*P* = 0.0199).

### Immunofluorescence and staining

**Fig. 4:**
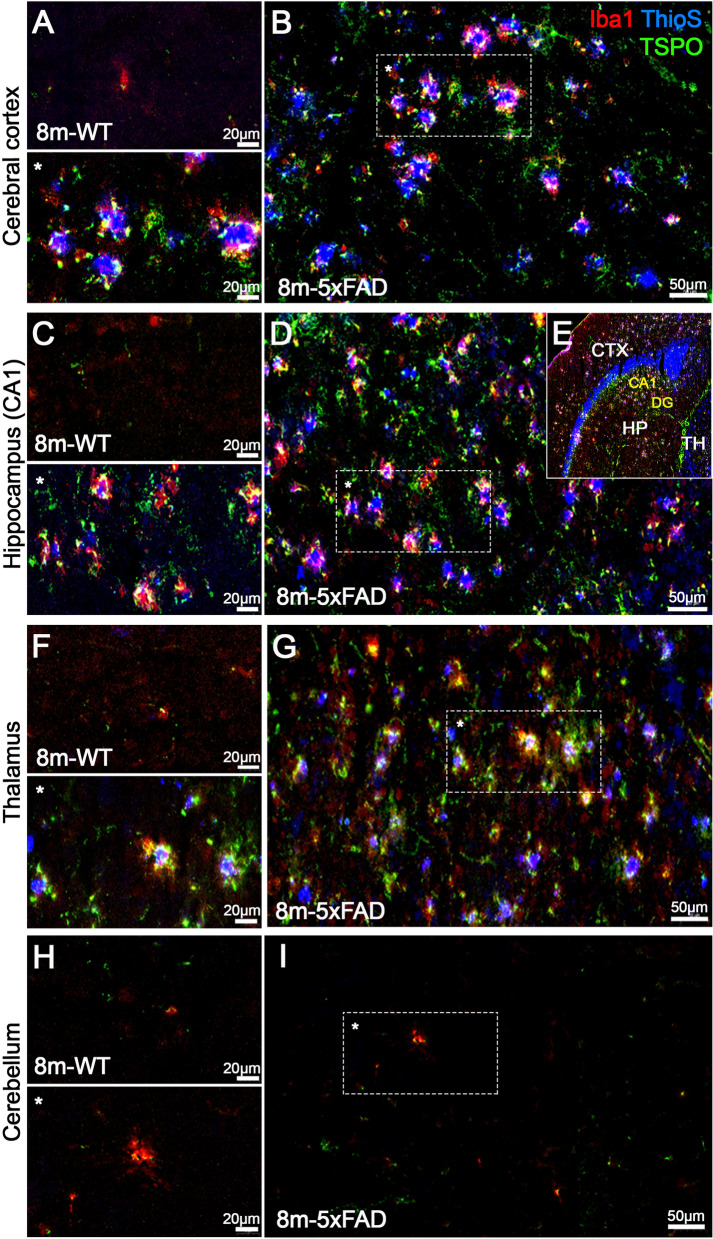
Staining of Iba1 (red), Thioflavin S (blue), and TSPO (green) in 8-month-old brains of WT (**A**, **C**, **F**, and **H**) and 5xFAD mice (**B**, **D**, **G**, and **I**) shown as merged channels. Representative brain mouse slide showing the cerebral regions evaluated with immunofluorescence labeling (CTX, cortex; HP, hippocampus (CA1 and DG dentate gyrus); TH, thalamus) (**E**)

**Fig. 5:**
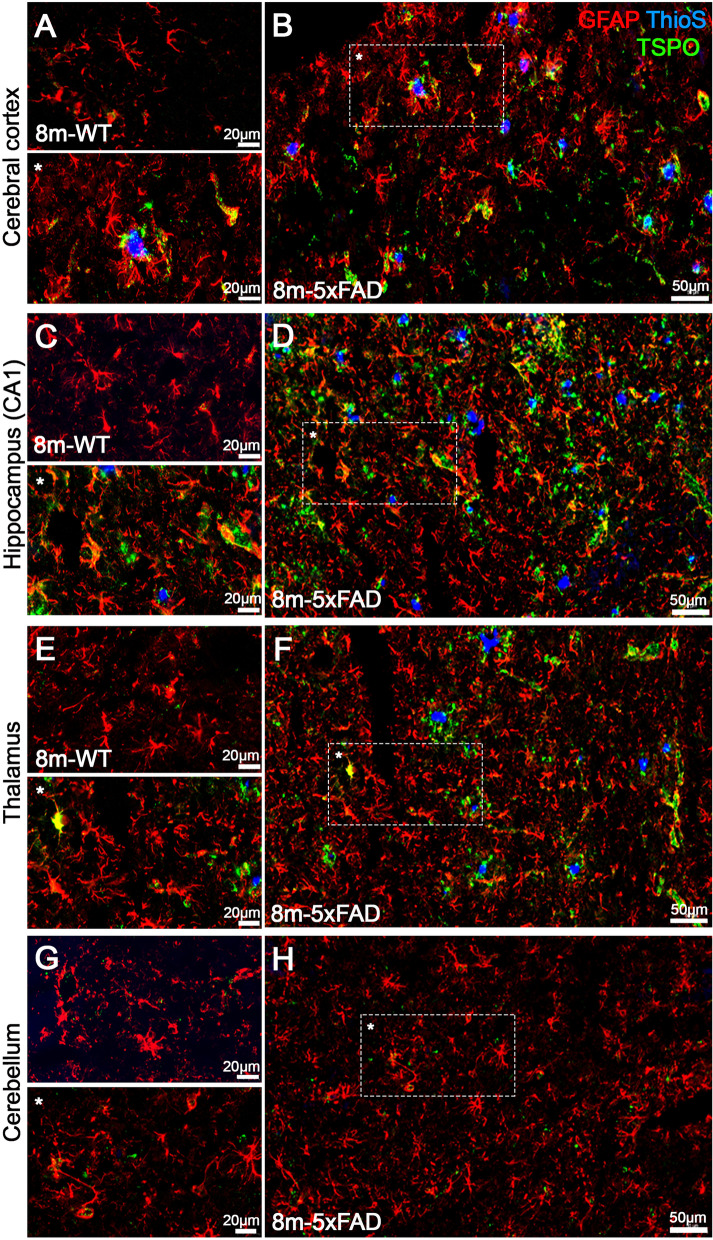
Staining of GFAP (red), Thioflavin S (blue), and TSPO (green) in 8-month-old brains of WT (**A**, **C**, **F**, and **H**) and 5xFAD mice (**B**, **D**, **G**, and **I**) shown as merged channels

Immunofluorescence staining showed TSPO overexpression in a heterogeneous population of glial and inflammatory cells such as microglia and infiltrated macrophages and astrocytes in 8- and 12-month-old brains of 5xFAD mice in comparison to WT (Figs. 4, 5, and S2). Iba1-positive cells (activated microglia/macrophages) overexpressing TSPO were observed surrounding Thioflavin S-positive fibrillar amyloid plaques in CTX, HIP, and THA of 5xFAD mice (in yellow and pink; Fig. 4B, D, and G). Conversely, WT mice showed low microglia/macrophage activation and TSPO expression in the different brain regions considered (Fig. 4A, C, and F). Likewise, astrocytes displayed an increase of the GFAP immunoreactivity expressing TSPO in CTX, HIP, and THA of 5xFAD mice (in yellow; Fig. 5B, D, and F). Unlike Iba1 and TSPO colocalization, GFAP-positive astrocytes expressing TSPO were not restricted to the surrounding area of Aβ plaque deposition. In addition, WT brains showed lower astrocytic reactivity and scarce TSPO expression in CTX, HIP, and THA (Fig. 5A, C, and E). Finally, CB in 5xFAD mice displayed low TSPO expression levels in comparison to other brain regions, supporting its usefulness as a reference region for PET analyses (Figs. 4I and 5H).

**Fig. 6:**
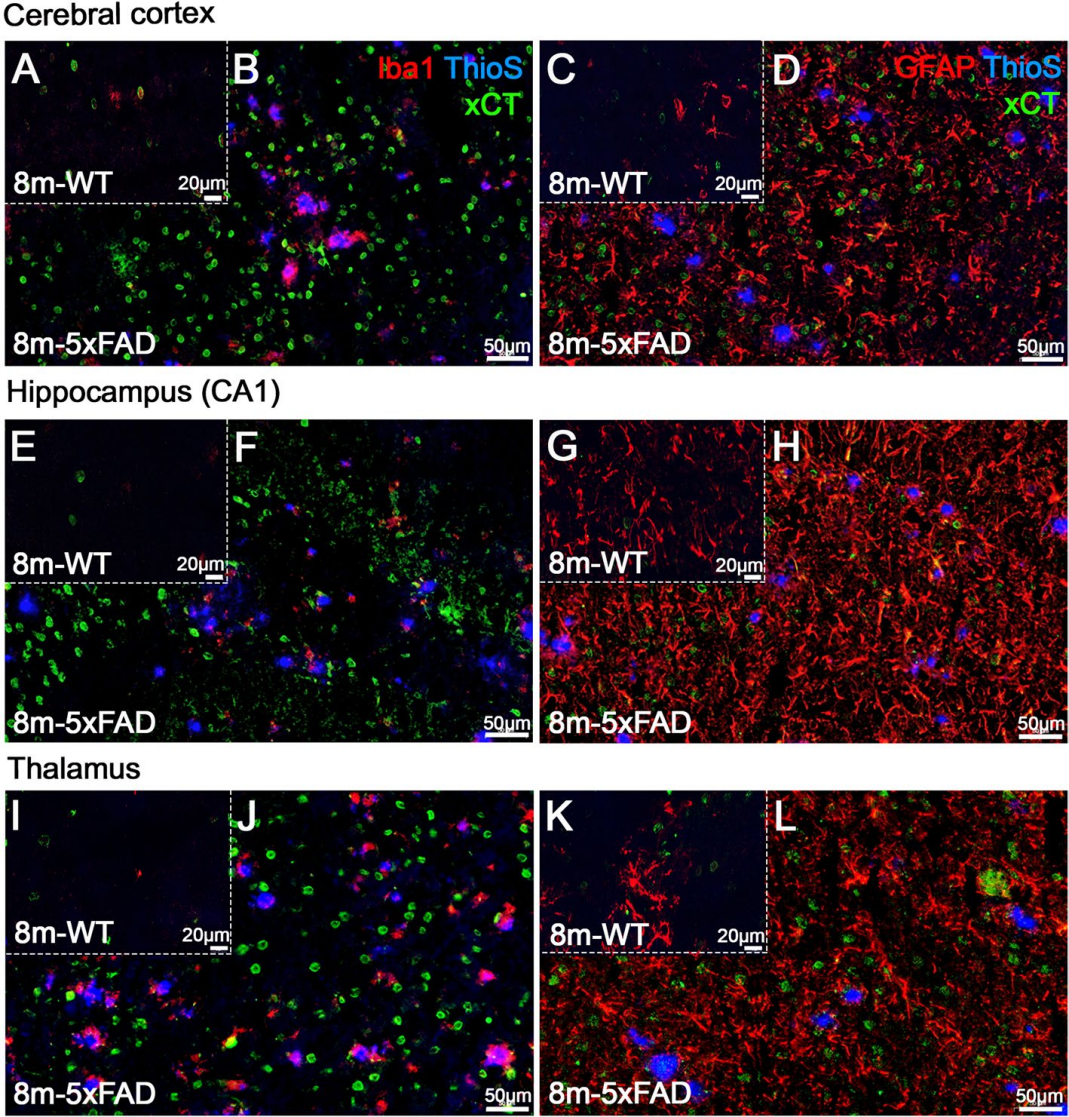
Staining of Iba1 or GFAP (red), Thioflavin S (blue), and xCT (green) in 8-month-old brains of WT (**A**, **C**, **E**, **G**, **I**, and **K**) and 5xFAD mice (**B**, **D**, **F**, **H**, **J**, and **L**) shown as merged channels

Immunofluorescence labeling of xCT showed an overexpression in 8- and 12-month-old brains of 5xFAD mice in comparison to WT (Figs. 6 and S3). These results showed an increase of xCT immunoreactivity in both non-glial cells and immune cells (Iba1- and GFAP-positive cells) in CTX, HIP, and THA of 5xFAD mice that were not colocalized with Thioflavin S-positive amyloid plaques (Fig. 6B, D, F, H, J, and L). Besides, these findings were in accordance with the 4-hydroxy-2-nonenal (4-HNE) labeling increase in the cerebral cortex and hippocampus observed in 12-month-old brains of 5xFAD mice in comparison to WT (Fig. 4S). The formation of aldehydic products such as 4-HNE) as a result of lipid peroxidation has been implicated in the etiology of pathological changes under oxidative stress as a key mediator of oxidative stress-induced cell death.

### MRI studies

**Fig. 7:**
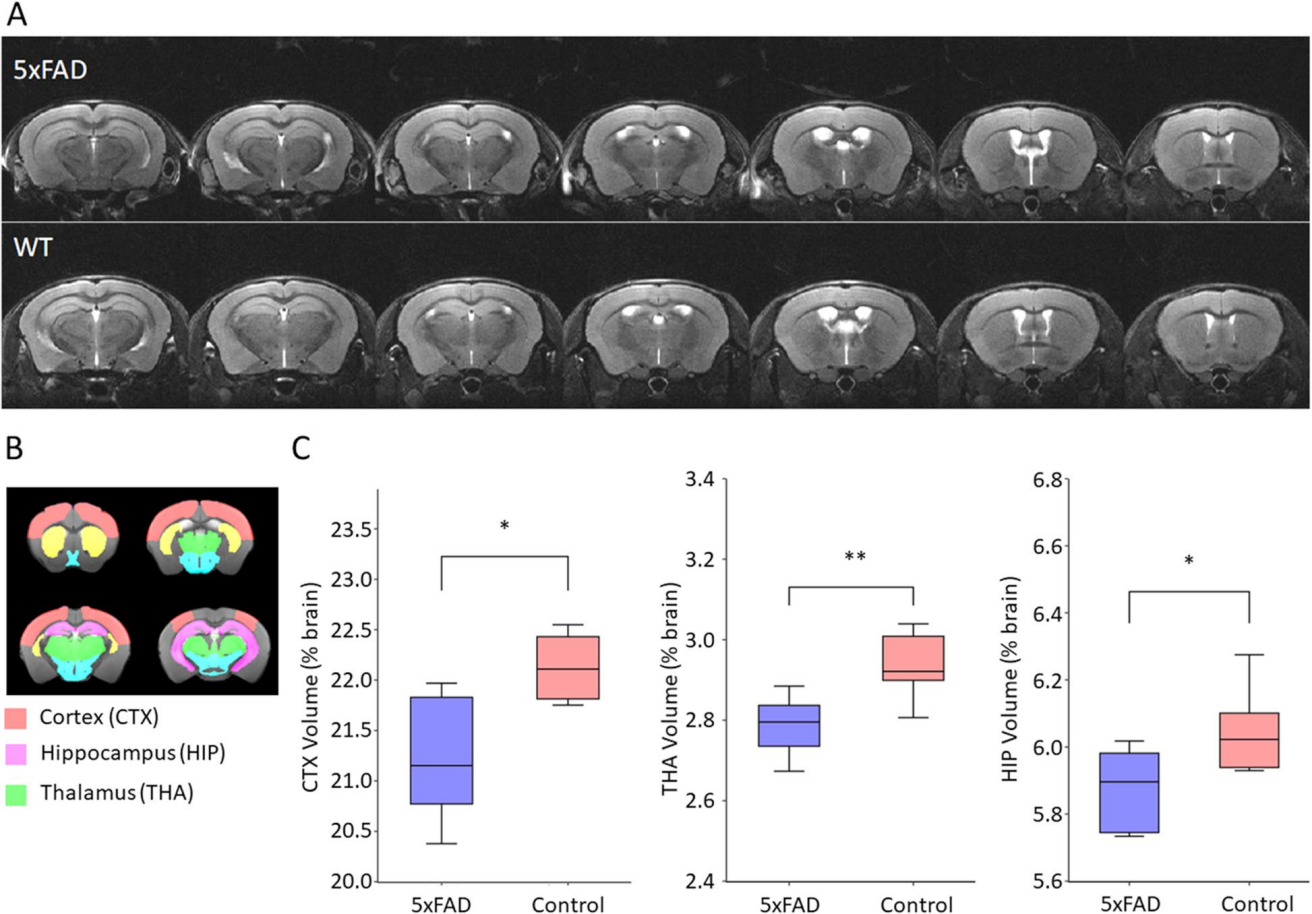
Volume measurements of different regions of interest in 12-month-old 5xFAD and control mice. A 7 consecutive planes (500 µm thickness) of the T2w images acquired for one 5xFAD and one control mice. B Delineation of the regions of interest considered for volumetric analysis, including the three regions that showed significant differences (cortex, CTX; thalamus, THA; and hippocampus, HIP). C Box plots of the normalized volumes (% total brain volume) for the ROIs that presented significant changes in volume at the level of ***P*** < 0.05 (*) or ***P*** < 0.01 (**) (cortex, CTX; thalamus, THA; and hippocampus, HIP)

Volumetric measurements of different brain regions (Fig. 7) and values of diffusion-related parameters (FA, fractional anisotropy; RD, radial diffusivity; AD, axial diffusivity; and MD, mean diffusivity) obtained from diffusion tensor imaging (DTI) studies were analyzed. Differences between controls and 5XFAD mice for multiple brain regions were calculated as % of change. A significant reduction in brain volume was observed in 5xFAD mice compared to controls in CTX (ΔV = −4,1%, *p* < 0.05), HIP (ΔV = −2.81%, *p* < 0.05), and THA (ΔV = −5.10%, *p* < 0.01) (Fig. 7C). Whole brain volumes of the transgenic model were not significantly different from controls (ΔV = −4.68 %). Microstructural alterations on brain tissue were also detected in the analysis of diffusion-related imaging parameters. Significant differences in radial diffusivity (RD) and/or fractional anisotropy (FA) were observed between 5xFAD and WT mice in subcortical (THA: ΔRD = 3.5%, *p* < 0.05) and cortical (CTX: ΔFA = −10.4%, *p* < 0.05; HIP: ΔRD = 4.6% *p* < 0.05) regions. Differences of these parameters in other regions and differences for other diffusion-related parameters such as axial diffusivity (AD) or mean diffusivity (MD) showed no significant differences for all regions.

## Discussion

TSPO protein overexpression and cysteine/glutamate antiporter gene are known to be associated with AD, due to their involvement in neuroinflammation and formation of reactive oxygen and nitrogen species (RONS)*.* For the purposes of this study, 5xFAD mice were chosen as they faithfully recapitulate AD-associated Aβ pathology, and the uptake of two radiotracers, [^18^F]DPA-714 and [^18^F]FSPG, was used as a surrogate for neuroinflammation (TSPO) and oxidative stress levels (system x_c_^-^), respectively. The results were compared to longitudinal PET imaging results with [^18^F]florbetaben obtained in the same mice as recently reported [29].

The use of a specific TSPO marker, [^18^F]DPA-714, was ruled appropriate to assess neuroinflammation in mice. IHC analysis showed low expression of TSPO in CB of 5xFAD mice (Figs. 4 and 5), suggesting that this brain region is an appropriate reference for the assessment of neuroinflammation through [^18^F]DPA-714-PET imaging. This is consistent with a previous study that selected CB as a reference region for [^18^F]DPA-714-PET imaging of a different animal model of AD [33]. Contrary to this previous study, our TACs in the whole brain did not show the presence of a plateau within the duration of the PET scans. This posed some doubts on the possibility of quantifying the results by determining the uptake ratio between the region of interest and CB. The results were therefore analyzed by Regional Logan Plot analysis using CB as the reference region and reported as DVRs. Contrary to the original Logan Plot analysis, Regional Logan Plot analysis does not require plasma sampling and allows the estimation of DVR (or non-displaceable binding potential, BP_ND_; calculated as BP_ND_ = DVR − 1) from reversible ligand-receptor PET studies.

Regarding the x_c_^-^ antiporter system, our animal model exhibited low but sufficient [^18^F]FSPG uptake into the brain. Contrarily to the case of [^18^F]DPA-714, no evident reference region could be selected for [^18^F]FSPG. The option of using the blood input function for kinetic modeling was also unfeasible, because the heart and major vessels, which have been previously used to determine image-derived input functions [34], were out of the FOV of the PET camera. The quantification was therefore based on averaging images obtained at the last three frames, to determine uptake values in each region, as described before [20]. The lack of an appropriate reference region disabled correction for deviations in the injected activity. Furthermore, a source of radioactivity outside of the brain was observed, likely due to uptake by immune cells in the meninges/subarachnoid space or a meningeal lymphatic vessel, as previously suggested [28]. This could result in radioactive signal spill-over into CB. All these factors reflected in higher intrasubject variability, which ultimately resulted in high standard deviation values. Despite these limitations, some conclusions could be made based on the obtained results.

Control mice exhibited a small but insignificant increase in [^18^F]DPA-714 and [^18^F]FSPG uptake in the brain over time up to 8 months, which indicated that TSPO and RONS did not evolve as a result of aging in these animals. In contrast, the longitudinal uptake of both radiotracers in the brain of diseased mice indicated that neuroinflammation and oxidative stress followed the trend observed for Aβ deposition, as indicated in our previous [^18^F]florbetaben-PET study [29]. Accordingly, the initial imaging session showed only slightly, non-significantly higher uptake of [^18^F]DPA-714 and [^18^F]FSPG in 5xFAD compared to WT mice. The difference increased as the disease progressed, at 5 and 8 months of age. Although both radiotracers showed increased uptake in CTX, HIP, and THA of 5xFAD compared to WT (brain regions which show increased beta-amyloid plaque burden at age > 3 months in this animal model [32]), the differences were notably higher for [^18^F]DPA-714. These findings were confirmed by qualitative immunofluorescence analysis. The brains of 8-month-old mice from this study were initially chosen, because no significant changes in radiotracer uptake occurred at later time points of the PET study. Additionally, PET image analysis could not account for changes in tissue uniformity and shrinkage, which would disable comparison between in vivo and ex vivo techniques. Ex vivo analysis showed that TSPO and xCT concentration increased in CTX, HIP, and THA of 5xFAD mice in comparison to age-matched controls, without notable differences among these brain regions. Furthermore, highly myelinated brain regions exhibited higher concentrations of microglia/infiltrated macrophages overexpressing TSPO, which could be the reason behind the notable increase of TSPO overexpression in THA observed in PET. On the other hand, xCT overexpression was observed in non-glial cells throughout the brain, suggesting that the increase of neuronal oxidative stress is associated with the overall increase of Aβ rather than with regional pathology. This also explained the absence of the reference region within the brain for PET image analysis. Additionally, higher oxidative stress levels induced lipid peroxidation and the subsequent accumulation of the 4 HNE in AD brains compared with WT.

[^18^F]DPA-714 uptake levels in 5xFAD mice continued to slightly increase in HIP and THA after the 8-month time point, but the uptake in CTX between 8 and 12 months remained constant. This is surprising, because more neuroinflammation is expected as the disease progresses. While this could be a specific characteristic of the disease or this animal model, a contribution of other factors, such as brain tissue shrinkage in some brain regions, might also be the cause for this phenomenon. Indeed, MRI analysis at 12 months of age showed cortical tissue shrinkage, a common feature in late-stage AD. Furthermore, DTI revealed extensive damage on brain microstructure, likely indicating neuronal damage. Of note, the inaccessibility of the PET-MRI camera prevented correct delineation of VOIs; therefore, the same brain atlas was used for all the mice, regardless of age. Although the brain size was corrected by MRI-CT co-registration, the brain tissue shrinkage could not be accounted for and is a possible reason behind these discrepancies.

**Fig. 8:**
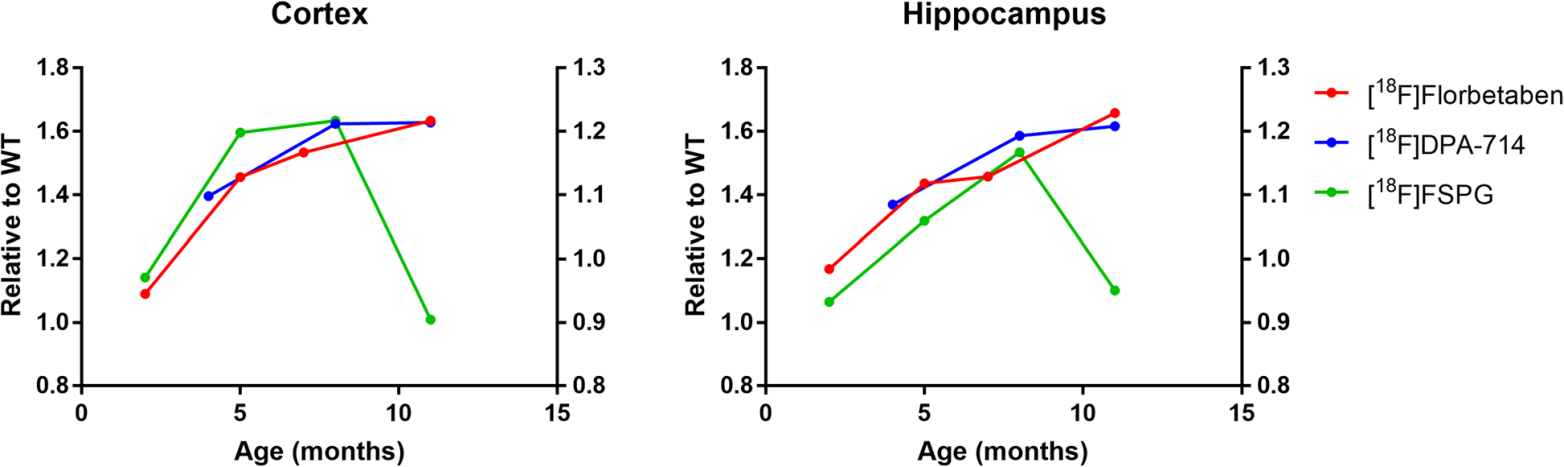
Tracer uptake for 5xFAD mice, relative to the uptake obtained for age-matched WT animals, in the cortex (CTX) and hippocampus (HC) at different ages and using different tracers. The red line (right ***Y*** axis), corresponding to [^18^F]florbetaben, represents age-matched 5xFAD/WT ratios of SUVr to the cerebellum; the blue line (right ***Y*** axis), corresponding to [^18^F]DPA-714, represents age-matched 5xFAD/WT ratios of DVR; the green line (left ***Y*** axis), corresponding to [^18^F]FSPG, represents age-matched 5xFAD/WT ratios of SUV

For comparative purposes among the different ligands, the evolution of radiotracer uptake in AD animals with respect to age-matched WT animals is depicted in Fig. 8.

In the figure, relative average values (SUVr to the cerebellum for [^18^F]florbetaben, obtained from [29]; SUV for [^18^F]FSPG; DVR for [^18^F]DPA-714) obtained both in CTX and HIP for each group and age are plotted. In both brain subregions, the profiles corresponding to [^18^F]florbetaben and [^18^F]DPA-714 follow the same trend, with a progressive increase over time that tends to stabilize after 8 months. Contrarily, the relative increased uptake for [^18^F]FSPG in 5xFAD animals seems to show an earlier, more abrupt onset between 2 and 4 months of age, to stabilize afterwards. Interestingly, relative values for this radiotracer drop to *ca.* 1 at the age of 11 months, which means that at this age, both WT and AD animals show similar tracer uptake. Noteworthy, this abrupt decrease is not due to a decrease in the uptake observed in 5xFAD animals, but to an increase in [^18^F]FSPG uptake in aged WT animals. The causes behind such an increase remain unclear and require further investigation. Still, the different time profile obtained for this tracer suggests that it could complement neuroinflammation and β-amyloid imaging biomarkers for more accurate longitudinal investigation of the pathophysiology of the disease and monitoring of AD progression.

### Study limitations

The study has one major limitation. As mentioned in the experimental, results, and discussion sections, the imaging studies were performed on two batches of animals (both batches comprising WT and 5xFAD animals). For the first batch of animals, and due to the high number of scans with other tracers ([^18^F]florbetaben and [^11^C]**4** to assess Aβ burden and butyrylcholinesterase expression, respectively) performed on the animals, [^18^F]FSPG and [^18^F]DPA-714 scans could be performed only at 2 and 12 months, respectively. To complete the longitudinal evaluation of neuroinflammation and oxidative stress, a second batch of animals was purchased to cover those ages not included in the first study. Despite this, the same species and strain were used, and the animals were treated following the same procedures. Additionally, resting times in between consecutive scans were respected. Hence, although evaluation of neuroinflammation and oxidative stress was not performed in the same individuals (this is, the study is not purely “longitudinal”) and paired analysis was not possible, we are positive that the results are conclusive and reliable.

The second limitation of the study is related to the capability of [^18^F]FSPG to cross the blood-brain barrier. Brain uptake of the radiotracer was low, and a source of radioactivity outside of the brain was observed, likely due to uptake by immune cells in the meninges/subarachnoid space or in meningeal lymphatic vessel. This, together with the lack of an evident reference region, made quantification difficult and resulted in high variability of the results. Despite significant differences could be observed, the development of radiotracers with better brain penetration and less off-target accumulation might solve the problem, although to the best of our knowledge, no tracers to better quantify the activity of the xCT system in the brain have been developed to date.

The third limitation of the study is the method used to delineate the different brain subregions. As mentioned in the experimental section, an atlas available in the π-MOD image analysis software was used to generate the VOIs covering the cortex, hippocampus, thalamus, and cerebellum. According to MRI results obtained at 12 months of age, a significant reduction in brain volume was observed in 5xFAD mice compared to controls in the cortex, hippocampus, and thalamus. This fact may pose a certain bias on the quantification of the radiotracer uptake in these regions, especially at advanced ages. Co-registration of the PET images with MRI anatomical images obtained with the same individuals at each time point may mitigate this error.

## Conclusions

In conclusion, [^18^F]DPA-714 and [^18^F]FSPG show prospect to monitor AD progression. TSPO overexpression and increased oxidative stress accompany Aβ accumulation, thus becoming potential in vivo diagnostic/prognostic tools for Aβ-associated neurodegeneration. The results support the involvement of neuroinflammatory processes and oxidative stress in Aβ pathology, adding to the pool of knowledge on AD disease mechanism and encouraging the expansion of AD diagnosis beyond the traditional AT(N) scheme.

## Supplementary Information

**Additional file 1. **Supporting information.

## Data Availability

The datasets used and/or analyzed during the current study are available from the corresponding author on reasonable request.

## Abbreviations

Aβ: Amyloid beta
AD: Alzheimer disease
BBB: Blood-brain barrier
CB: Cerebellum
CT: Computerized tomography
CTX: Cortex
DTI: Diffusion tensor imaging
DVR: Distribution volume ratio
FA: Fractional anisotropy
FBP: Filtered back projection
FOV: Field of view
HIP: Hippocampus
MRI: Magnetic resonance imaging
PET: Positron emission tomography
RD: Radial diffusivity
ROI: Region of interest
SD: Standard deviation
SUV: Standard uptake value
T_1_W: T_1_-weighed
TAC: Time-activity curve
THA: Thalamus
TSPO: Translocator protein (18 kDa)
VOI: Volume of interest
WT: Wild type

## References

[1] Global, regional, and national burden of Alzheimer’s disease and other dementias, 1990-2016: a systematic analysis for the Global Burden of Disease Study 2016. Lancet Neurol*.* 2019;18:88–106. 10.1016/S1474-4422(18)30403-4 PMC6291454 30497964

[2] Dunn B, Stein P, Cavazzoni P (2021). Approval of aducanumab for Alzheimer disease—the FDA’s perspective. JAMA Intern Med.

[3] Selkoe DJ (2019). Alzheimer disease and aducanumab: adjusting our approach. Nat Rev Neurol..

[4] Tampi RR, Forester BP, Agronin M (2021). Aducanumab: evidence from clinical trial data and controversies. Drugs Context..

[5] Selkoe DJ, Hardy J (2016). The amyloid hypothesis of Alzheimer’s disease at 25 years. EMBO Mol Med..

[6] Goedert M, Spillantini MG, Cairns NJ, Crowther RA (1992). Tau proteins of Alzheimer paired helical filaments: abnormal phosphorylation of all six brain isoforms. Neuron.

[7] Forner S, Baglietto-Vargas D, Martini AC, Trujillo-Estrada L, LaFerla FM (2017). Synaptic impairment in Alzheimer’s disease: a dysregulated symphony. Trends Neurosci.

[8] Jack CR, Bennett DA, Blennow K (2018). NIA-AA Research Framework: toward a biological definition of Alzheimer’s disease. Alzheimers Dement..

[9] Koizumi K, Wang G, Park L (2016). Endothelial dysfunction and amyloid-β-induced neurovascular alterations. Cell Mol Neurobiol..

[10] Walters A, Phillips E, Zheng R, Biju M, Kuruvilla T (2016). Evidence for neuroinflammation in Alzheimer’s disease. Prog Neurol Psychiatry.

[11] Smith MA, Rottkamp CA, Nunomura A, Raina AK, Perry G (2000). Oxidative stress in Alzheimer’s disease. Biochim Biophys Acta..

[12] Chen L, Liu B (2017). Relationships between stress granules, oxidative stress, and neurodegenerative diseases. Oxid Med Cell Longev..

[13] Oakley H, Cole SL, Logan S (2006). Intraneuronal beta-amyloid aggregates, neurodegeneration, and neuron loss in transgenic mice with five familial Alzheimer’s disease mutations: potential factors in amyloid plaque formation. J Neurosci..

[14] Cheignon C, Tomas M, Bonnefont-Rousselot D, Faller P, Hureau C, Collin F (2018). Oxidative stress and the amyloid beta peptide in Alzheimer’s disease. Redox Biol..

[15] Akiyama H, Barger S, Barnum S (2000). Inflammation and Alzheimer’s disease. Neurobiol Aging..

[16] Kreisl WC, Kim M-J, Coughlin JM, Henter ID, Owen DR, Innis RB (2020). PET imaging of neuroinflammation in neurological disorders. Lancet Neurology..

[17] Narayanaswami V, Dahl K, Bernard-Gauthier V, Josephson L, Cumming P, Vasdev N (2018). Emerging PET radiotracers and targets for imaging of neuroinflammation in neurodegenerative diseases: outlook beyond TSPO. Mol Imaging..

[18] Van Camp N, Lavisse S, Roost P, Gubinelli F, Hillmer A, Boutin H. TSPO imaging in animal models of brain diseases. Eur J Nucl Med Mol Imaging. 2021;49(1):77–109. 10.1007/s00259-021-05379-z PMC8712305 34245328

[19] Martin A, Boisgard R, Theze B (2010). Evaluation of the PBR/TSPO radioligand [(18)F]DPA-714 in a rat model of focal cerebral ischemia. J Cereb Blood Flow Metab..

[20] Domercq M, Szczupak B, Gejo J (2016). PET imaging with [(18)F]FSPG evidences the role of system xc(-) on brain inflammation following cerebral ischemia in rats. Theranostics..

[21] Mirzaei N, Tang SP, Ashworth S (2016). In vivo imaging of microglial activation by positron emission tomography with [(11)C]PBR28 in the 5XFAD model of Alzheimer's disease. Glia.

[22] Chaney A, Williams SR, Boutin H (2019). In vivo molecular imaging of neuroinflammation in Alzheimer’s disease. J Neurochem.

[23] Bridges R, Lutgen V, Lobner D, Baker DA (2012). Thinking outside the cleft to understand synaptic activity: contribution of the cystine-glutamate antiporter (System xc-) to normal and pathological glutamatergic signaling. Pharmacol Rev..

[24] Koglin N, Mueller A, Berndt M, et al. Specific PET imaging of xC- transporter activity using a^1^^8^F-labeled glutamate derivative reveals a dominant pathway in tumor metabolism. Clin Cancer Res*.* 2011;17:6000–11. 10.1158/1078-0432.CCR-11-0687 21750203

[25] Zaragoza R (2020). Transport of amino acids across the blood-brain barrier. Front Physiol..

[26] Patel SA, Warren BA, Rhoderick JF, Bridges RJ (2004). Differentiation of substrate and non-substrate inhibitors of transport system xc(-): an obligate exchanger of L-glutamate and L-cystine. Neuropharmacology.

[27] Park SY, Mosci C, Kumar M (2020). Initial evaluation of (4S)-4-(3-[(18)F]fluoropropyl)-L-glutamate (FSPG) PET/CT imaging in patients with head and neck cancer, colorectal cancer, or non-Hodgkin lymphoma. EJNMMI Res..

[28] Hoehne A, James ML, Alam IS (2018). [(18)F]FSPG-PET reveals increased cystine/glutamate antiporter (xc-) activity in a mouse model of multiple sclerosis. J Neuroinflammation..

[29] Rejc L, Gomez-Vallejo V, Joya A (2021). Longitudinal evaluation of a novel BChE PET tracer as an early in vivo biomarker in the brain of a mouse model for Alzheimer disease. Theranostics..

[30] Ismeurt C, Giannoni P, Claeysen S. Chapter 13 - The 5×FAD mouse model of Alzheimer’s disease. In: Martin CR, Preedy VR, eds. Diagnosis and Management in Dementia: Academic Press; 2020:207–221.

[31] Pulagam KR, Colas L, Padro D (2017). Evaluation of the novel TSPO radiotracer [(18)F] VUIIS1008 in a preclinical model of cerebral ischemia in rats. EJNMMI Res..

[32] Oblak AL, Lin PB, Kotredes KP, et al. Comprehensive evaluation of the 5XFAD mouse model for preclinical testing applications: a MODEL-AD Study. Front Aging Neurosci. 2021;13:713726. 10.3389/fnagi.2021.713726. 10.3389/fnagi.2021.713726 PMC8346252 34366832

[33] Takkinen JS, Lopez-Picon FR, Al Majidi R (2017). Brain energy metabolism and neuroinflammation in ageing APP/PS1-21 mice using longitudinal (18)F-FDG and (18)F-DPA-714 PET imaging. J Cereb Blood Flow Metab..

[34] Perez-Campana C, Gomez-Vallejo V, Puigivila M (2014). Assessing lung inflammation after nanoparticle inhalation using 2-deoxy-2-[18F]fluoro-D-glucose positron emission tomography imaging. Mol Imaging Biol..

