# Supplementary material for: Longitudinal evaluation of neuroinflammation and oxidative stress in a mouse model of Alzheimer disease using positron emission tomography
Source: Alzheimers Res Ther. 2022 Jun 9;14:80. doi: 10.1186/s13195-022-01016-5 (PMC9178858; doi:10.1186/s13195-022-01016-5)
Supplement: Supplementary file 1 — Additional file 1. Supporting information. [file 13195_2022_1016_MOESM1_ESM.docx]

# SUPPORTING INFORMATION

Longitudinal evaluation of neuroinflammation and oxidative stress in a mouse model of Alzheimer Disease using Positron Emission Tomography

Luka Rejc,^a,*^ Vanessa Gómez-Vallejo,^a^ Ana Joya,^a,b^ Gemma Arsequell,^c^ Ander Egimendia,^a^ Pilar Castellnou,^a^ Xabier Ríos-Anglada,^a^ Unai Cossío,^a^ Zuriñe Baz,^a^ Leyre Iglesias,^b^ Estibaliz Capetillo-Zarate,^d,e^ Pedro Ramos-Cabrer,^a,e^ Abraham Martin,^b,e^ Jordi Llop^a,†^

^a^ CIC biomaGUNE, Basque Research and Technology Alliance (BRTA), Paseo Miramon 182, 20014, San Sebastian, Spain.

^b^ Laboratory of Neuroimaging and biomarkers of inflammation, Achucarro Basque Center for Neuroscience, Science Park UPV/EHU, Sede building B. Sarriena, 48940 Leioa, Spain

^c^ Institut de Química Avançada de Catalunya (IQAC), Spanish Council of Scientific Research (IQAC-CSIC), 08034 Barcelona, Spain

^d^ Faculty of Medicine and Nursery, University of the Basque Country UPV/EHU, Achucarro Basque Center for Neuroscience and CIBERNED, Barrio Sarriena S/N, 48940 Leioa, Spain

^e^ IKERBASQUE, Basque Foundation for Science, 48009 Bilbao, Spain

Corresponding authors

Luka Rejc: [rejc.luka87@](mailto:rejc.luka87@)gmail.com; +18588680966

Jordi Llop: [jllop@cicbiomagune.es](mailto:jllop@cicbiomagune.es); +34943005333

# Materials and Methods

## Reagents and general methods

Chemicals and solvents were obtained from commercial sources (i.e., Acros Organics, Alfa Aesar, Fluka, Merck, Sigma-Aldrich, TCI Europe) and were used without purification unless otherwise stated. Ultrapure water (resistivity > 18MΩcm) was generated using a Milli-Q system (Millipore, Bedford, MA, USA).

Fluorine-18 (^18^F; T_1/2_ = 109.8 min) was cyclotron-produced (Cyclone-18/9, IBA, Louvain-la-Neuve, Belgium) as [^18^F]fluoride by proton irradiation of an ^18^O-enriched (98%) water target (2.9 mL) via the ^18^O(p,n)^18^F nuclear reaction.

## Chemistry and radiochemistry

Production of [^18^F]DPA-714

The synthesis of *N*,*N*-diethyl-2-(2-(4-(2-fluoroethoxy)phenyl)-5,7-dimethylpyrazolo[1,5-a]pyrimidin-3-yl)acetamide ([^18^F]DPA-714) was performed using a TRACERlab FX_FN_ synthesis module (GE Healthcare), based on a one-step procedure, as previously described [[1](#_ENREF_1)]. Briefly, once transferred into a dedicated (ventilated and lead-shielded) hot-cell, [^18^F]fluoride was first trapped on a pre-conditioned Sep-Pak® Accell Plus QMA Light cartridge (Waters, Milford, MA, USA), then eluted from the cartridge with a solution of Kryptofix K_2.2.2/_K_2_CO_3_ in a mixture of water and acetonitrile. A solution containing the appropriate precursor for labeling (*N*,*N*-diethyl-2-(2-(4-(2-toluenesulfonyloxyethoxy)phenyl)-5,7-dimethylpyrazolo [1,5-a]pyrimidin-3-yl)acetamide, 4 mg) in dimethylsulfoxide (0.7 mL) was added and the mixture heated at 165 °C for 5 min. The reactor was then cooled at room temperature, the reaction crude diluted with a mixture of acetonitrile and water (2/1, 3 mL), and purified by high performance liquid chromatography (HPLC) using a Nucleosil 100-7 C18 column (Macherey-Nagel, Düren, Germany) as stationary phase and 0.1M aqueous ammonium formate solution (pH = 3.9)/acetonitrile (30/70) as the mobile phase at a flow rate of 7 mL/min. The desired fraction (10–11 min) was collected, diluted with water (20 mL), and the radiotracer was retained on a C-18 cartridge (Sep-Pak® Light, Waters, Milford, MA, USA) and further eluted with ethanol (1 mL). The ethanol solution was finally reconstituted with saline solution (9 mL). Filtration through a 0.22 μm filter yielded the final solution, ready for injection. Radiochemical yields (non-decay corrected) were in the range 8–13% and radiochemical purity was always >95% at the time of injection. Chemical and radiochemical purity and molar activity were determined by HPLC using an Agilent 1200 Series system equipped with a radioactivity detector (Gabi, Raytest) and a variable wavelength detector (λ = 254 nm) connected in series. A RP-C18 column (Mediterranea Sea18, 150 × 4.6 mm, 5 μm; Teknokroma, Spain) was used as the stationary phase and ammonium formate buffer (0.1M, pH = 3.9; A)/acetonitrile (40/60, v/v; B) as the mobile phase at a flow rate of 1.5 mL/min, with the following gradient: t=0 min, 90%A; t=1 min, 90%A; t=10min, 30%A; t=17min, 30%A; t=19min, 90%A; t=22min, 90%A (t_R_ = 9.0 min).

Production of [^18^F]FSPG

The synthesis of (4S)-4-[3-[^18^F]fluoropropyl]-*L*-glutamate ([^18^F]FSPG) was performed using a TRACERlab FX_FN_ synthesis module (GE Healthcare), based on a two-step procedure, as previously described [[2](#_ENREF_2)]: i) ^18^F-fluorination of the appropriately protected precursor for labeling (di-tert-butyl(2S,4S)-2-tert-butoxycarbonylamino-4-(3-nitrophenylsulfonyloxy-propyl)-pentanedioate), followed by ii) removal of the protective groups. Briefly, once transferred into a dedicated (ventilated and lead-shielded) hot-cell, [^18^F]fluoride was first trapped on a pre-conditioned Sep-Pak® Accell Plus QMA Light cartridge (Waters, Milford, MA, USA), then eluted from the cartridge with a solution of Kryptofix K_2.2.2_/K_2_CO_3_ in a mixture of water and acetonitrile. A solution of the precursor for labeling (5 mg) in acetonitrile (1 mL) was then added and the mixture heated at 80 ºC for 10 min. The crude reaction mixture was then diluted with a solution of acetonitrile and water (1/1, 2 mL) and purified by high performance liquid chromatography (HPLC) using a Nucleosil 100-7 C18 column (Macherey-Nagel, Düren, Germany) as stationary phase and water/acetonitrile (30/70) as the mobile phase at a flow rate of 8 mL/min. The desired fraction (11–13 min) was collected and diluted with water (50 mL). The labeled specie was retained on a C-18 cartridge (Sep-Pak® Light, Waters, Milford, MA, USA) and further eluted with ethanol (1 mL). This solution was treated with 0.5 ml of 4M HCl (500 μL); after hydrolysis, the solution was neutralized with 2N NaOH (650 μL) and phosphate buffer solution (PBS, pH = 7.4, 3 mL). After filtration through a 0.22 μm filter, the radiotracer was ready for injection. Radiochemical yields (decay-corrected) were in the range 30–35% and radiochemical purity was always >93% at the time of injection. Radiochemical purity was determined by HPLC using an Agilent 1200 Series system equipped with a radioactivity detector (Gabi, Raytest) and a variable wavelength detector (λ = 254 nm) connected in series. A RP-C18 column (Eclipse XDB C18, 150 × 4.6 mm, 5 μm; Agilent) was used as the stationary phase and 0.1% trifluoroacetic acid (TFA) in water (A)/0.1% TFA in acetonitrile (B) as the mobile phase at a flow rate of 1.0 mL/min, with the following gradient: t=0 min, 97%A; t=2 min, 97%A; t=10min, 10%A; t=15min, 10%A; t=18min, 97%A; t=20min, 97%A (t_R_ = 5.2 min).

## Animals and animal studies

Animal handling was conducted in accordance with the European Council Directive 2010/63/UE. All experimental procedures were approved by the Ethical Committee at CIC biomaGUNE and local authorities (authorization number: PRO-AE-SS-095).

Amyloid beta overexpressing female transgenic hemizygous 5xFAD mice (B6SJL-Tg(APPSwFlLon,PSEN1*M146L*L286V)6799Vas/Mmjax) and control female WT C57BL/6J × SJL/J F1 mice were obtained from The Jackson Laboratory (Bar Harbor, ME, USA) in two batches (batch 1: n(AD) = 10, n(WT) = 8; batch 2: n(AD) = 13, n(WT) = 10). Animals arrived to CIC biomaGUNE at the age of 10 weeks and were imaged at different time points. Imaging was performed during light phase of the light–dark cycle. Representative animals of each group were sacrificed at 8-months of age, and the brain harvested for *ex vivo* studies.

PET-CT longitudinal brain studies

Longitudinal brain studies were performed using an eXplore Vista-CT small animal PET-CT system (GE Healthcare, WI, USA). In all cases, anesthesia was induced with 3.0–5.0% isoflurane in pure oxygen and maintained during imaging studies with 1.5–2.0% isoflurane in pure oxygen. Transgenic 5xFAD and their age-matched WT control mice of the same genetic background were injected intravenously with [^18^F]DPA-714 (see Table S2 for injected activitites; injected volume: 100–150 µL; 4, 8, and 12 months of age), [^18^F]FSPG (see Table S2 for injected activities; injected volume: 100–150 µL; 2, 5, 8, and 12 months of age). Dynamic PET images were acquired in one bed position, with the brain centered in the middle of the field of view (FOV) to acquire the dynamic distribution in the brain in the energy range 400–700 keV (frames: 5 × 5 s, 5 × 10 s, 5 × 30 s, 5 × 60 s, 4 × 120 s, 4 × 240 s, 4 × 400s; total duration: 59.4 min) starting immediately after administration of the radiotracer. CT scans were acquired immediately after each PET acquisition (X-Ray energy: 40 kV, intensity: 140 µA). PET images were reconstructed using filtered back projection (FBP) applying random, scatter, and attenuation corrections.

For image analysis, PET images were co-registered with an MRI template (M. Mirrione-T2, available in π-MOD software analysis tool) and different brain regions, namely the cortex (CTX), the hippocampus (HIP), the thalamus (THA) and the cerebellum (CB) were automatically delineated. A volume of interest (VOI) was also placed on the whole brain (WB) to determine average pharmacokinetics of the radiotracer in all brain regions. In case of [^18^F]DPA-714, CB was selected as reference region, as previously reported [[3](#_ENREF_3)], and images were analyzed using Regional Logan Plot analysis to determine the distribution volume ratio (DVR). Where no reference region was available (in case of [^18^F]FSPG), values were reported as uptake values standardized to the animal weight (SUV) to account for differences in animal sizes between the two mice groups. The uptake values were determined by averaging the signal in the last 20-min of the dynamic PET scan.

For [^18^F]DPA-714 and [^18^F]FSPG image representation, the signal obtained in the last 20 min of the dynamic PET scan was first averaged for all the animals. For [^18^F]DPA-714 the values in different brain regions were divided, voxel-by-voxel, with values determined in CB to obtain images representing standardized uptake at the end of each PET scan for individual animals. For [^18^F]FSPG, the uptake values in all brain regions were normalized to the injected dose. All the images of one animal group (5xFAD and WT mice) at individual time points were averaged and presented as image slices of different brain regions.

The protocol for [^18^F]florbetaben PET imaging and image analysis is described in our previous publication [[4](#_ENREF_4)]. Of note, the same animal batches were used to inject all radiotracers (see Table S1); therefore, to comply with animal regulations and practices for animal well-being, and to ensure sufficient time for the animals to recover, the injections were spaced apart and the time-points for the three radiotracers could not always be matched.

**Table S1.** Imaging studies performed on WT and 5xFAD animals. Results obtained with [^11^C]**4** (butyrylcholinesterase inhibitor) and [^18^F]florbetaben have been already reported [[4](#_ENREF_4)]; ▲ [^18^F]FSPG; ● [^18^F]DPA-714; ■ [^18^F]florbetaben; ○ [^11^C]**4**.

|  |  | **Age (months)** | | | | | | | |
| --- | --- | --- | --- | --- | --- | --- | --- | --- | --- |
| WT batch 1 | **Animal #** | **2** | **4** | **5** | **6** | **8** | **10** | **11** | **12** |
|  | WT01 |  | ○ | ■ | ○ | ■○ | ○ |  |  |
|  | WT02 | ▲ | ○ | ■ | ○ | ■○ | ○ | ■ | ●○ |
|  | WT03 |  | ○ | ■ | ○ | ■○ | ○ | ■ | ●○ |
|  | WT04 | ▲ | ○ | ■ | ○ | ■○ | ○ | ■ | ●○ |
|  | WT05 |  | ○ | ■ | ○ | ■○ | ○ | ■ | ○ |
|  | WT06 |  | ○ | ■ | ○ | ■○ | ○ | ■ |  |
|  | WT07 | ▲ | ○ | ■ | ○ | ■ | ○ | ■ | ●○ |
|  | WT08 |  | ○ | ■ | ○ | ○ | ○ | ■ | ●○ |
|  | WT09 | ▲ | ○ |  | ○ | ○ |  | ■ | ○ |
|  | WT10 |  | ○ |  |  | ○ |  |  |  |
| WT batch 2 | WT24 | ■ | ● | ▲ |  | ▲● |  |  | ▲ |
|  | WT25 | ■ | ● | ▲ |  | ▲● |  |  | ▲ |
|  | WT26 | ■ |  |  |  |  |  |  | ▲ |
|  | WT27 | ■ | ● | ▲ |  | ▲● |  |  | ▲ |
|  | WT28 | ■ | ● | ▲ |  | ▲● |  |  | ▲ |
|  | WT29 | ■ | ● | ▲ |  | ▲● |  |  | ▲ |
|  | WT30 | ■ |  | ▲ |  | ▲● |  |  | ▲ |
|  | WT31 | ■ | ● | ▲ |  | ▲● |  |  | ▲ |

|  |  | **Age (months)** | | | | | | | |
| --- | --- | --- | --- | --- | --- | --- | --- | --- | --- |
| 5xFAD batch 1 | **Animal #** | **2** | **4** | **5** | **6** | **8** | **10** | **11** | **12** |
|  | AD11 | ▲ | ○ | ■ | ○ | ■○ | ○ | ■ | ●○ |
|  | AD12 |  | ○ | ■ | ○ | ■○ | ○ |  |  |
|  | AD13 |  | ○ | ■ | ○ | ■○ | ○ | ■ | ○ |
|  | AD14 |  | ○ | ■ | ○ | ■○ | ○ | ■ | ●○ |
|  | AD15 | ▲ | ○ | ■ | ○ | ■○ | ○ | ■ | ●○ |
|  | AD16 |  | ○ | ■ | ○ | ■○ | ○ | ■ | ○ |
|  | AD17 |  | ○ | ■ | ○ | ■○ | ○ | ■ | ○ |
|  | AD18 | ▲ | ○ | ■ | ○ | ○ | ○ | ■ | ●○ |
|  | AD19 | ▲ | ○ |  | ○ |  |  | ■ | ● |
|  | AD20 |  | ○ |  | ○ |  |  | ■ |  |
|  | AD21 | ▲ | ○ |  |  |  |  |  | ●○ |
|  | AD22 | ▲ | ○ |  |  |  |  |  | ○ |
|  | AD23 |  | ○ |  |  |  |  |  | ○ |
| 5xFAD batch 2 | AD32 | ■ | ● | ▲ |  | ▲● |  |  | ▲ |
|  | AD33 | ■ | ● | ▲ |  | ▲● |  |  | ▲ |
|  | AD34 | ■ | ● | ▲ |  | ▲● |  |  | ▲ |
|  | AD35 | ■ |  |  |  |  |  |  | ▲ |
|  | AD36 | ■ | ● | ▲ |  | ● |  |  | ▲ |
|  | AD37 | ■ | ● | ▲ |  | ▲● |  |  | ▲ |
|  | AD38 | ■ | ● | ▲ |  | ▲● |  |  | ▲ |
|  | AD39 | ■ |  | ▲ |  | ▲● |  |  | ▲ |
|  | AD40 | ■ |  | ▲ |  | ▲● |  |  | ▲ |
|  | AD41 |  |  | ▲ |  | ▲● |  |  | ▲ |

**Table S2.** Amount of radioactivity (in MBq) injected to each animal for [^18^F]DPA-714 and [^18^F]FSPG PET studies. Blank means that the animal was not studied with the tracer at that time point.

| **[^18^F]DPA-WT animals** | | | |
| --- | --- | --- | --- |
| **Animal #** | **4 months** | **8 months** | **12 months** |
| WT02 |  |  | 15.3 |
| WT03 |  |  | 15.4 |
| WT04 |  |  | 14.0 |
| WT07 |  |  | 17.2 |
| WT08 |  |  | 9.5 |
| WT24 | 12.8 | 15.9 |  |
| WT25 | 15.8 | 9.1 |  |
| WT27 | 12.7 | 11.7 |  |
| WT28 | 16.4 | 9.4 |  |
| WT29 | 8.3 | 12.1 |  |
| WT30 |  | 16.4 |  |
| WT31 | 14.9 | 19.2 |  |
| AVG | 13.5 | 13.4 | 14.3 |
| SDEV | 3.0 | 3.8 | 2.9 |

| **[^18^F]DPA-AD animals** | | | |
| --- | --- | --- | --- |
| **Animal #** | **4 months** | **8 months** | **12 months** |
| AD11 |  |  | 10.4 |
| AD14 |  |  | 14.1 |
| AD15 |  |  | 14.9 |
| AD18 |  |  | 16.7 |
| AD19 |  |  | 12.8 |
| AD21 |  |  | 13.8 |
| AD32 | 7.0 | 10.2 |  |
| AD33 | 12.4 | 13.2 |  |
| AD34 | 11.0 | 9.8 |  |
| AD36 | 15.3 | 14.5 |  |
| AD37 | 11.0 | 23.9 |  |
| AD38 | 10.0 | 18.4 |  |
| AD39 |  | 11.4 |  |
| AD40 |  | 20.5 |  |
| AD41 |  | 11.8 |  |
| AVG | 11.1 | 14.9 | 13.8 |
| SDEV | 2.7 | 5.0 | 2.1 |

| **[^18^F]FSPG-WT animals** | | | | |
| --- | --- | --- | --- | --- |
| **Animal #** | **2 months** | **5 months** | **8 months** | **12 months** |
| WT02 | 16.4 |  |  |  |
| WT04 | 16.2 |  |  |  |
| WT07 | 13.8 |  |  |  |
| WT09 | 10.8 |  |  |  |
| WT24 |  | 10.8 | 13.0 | 12.4 |
| WT25 |  | 12.9 | 11.2 | 9.8 |
| WT26 |  |  |  | 9.1 |
| WT27 |  | 8.3 | 12.9 | 14.2 |
| WT28 |  | 26.7 | 9.6 | 11.4 |
| WT29 |  | 18.8 | 6.0 | 8.3 |
| WT30 |  | 12.5 | 10.3 | 12.3 |
| WT31 |  | 17.3 | 10.5 | 14.5 |
| AVG | 14.3 | 15.3 | 10.5 | 11.5 |
| SDEV | 2.6 | 6.2 | 2.3 | 2.3 |

| **[^18^F]FSPG-AD animals** | | | | |
| --- | --- | --- | --- | --- |
| **Animal #** | **2 months** | **5 months** | **8 months** | **12 months** |
| AD11 | 11.4 |  |  |  |
| AD15 | 13.4 |  |  |  |
| AD18 | 16.1 |  |  |  |
| AD19 | 9.1 |  |  |  |
| AD21 | 13.1 |  |  |  |
| AD22 | 7.6 |  |  |  |
| AD32 |  | 14.3 | 14.3 | 15.7 |
| AD33 |  | 14.9 | 10.4 | 15.2 |
| AD34 |  | 8.8 | 11.0 | 16.2 |
| AD35 |  |  |  | 14.5 |
| AD36 |  | 15.0 |  | 11.4 |
| AD37 |  | 17.5 | 5.1 | 14.2 |
| AD38 |  | 18.8 | 3.5 | 10.4 |
| AD39 |  | 9.1 | 9.5 | 12.7 |
| AD40 |  | 17.4 | 8.7 | 6.1 |
| AD41 |  | 16.4 | 9.4 | 11.7 |
| AVG | 11.8 | 14.7 | 9.0 | 12.8 |
| SDEV | 3.1 | 3.6 | 3.4 | 3.1 |

Magnetic resonance imaging

MR imaging was performed as a single imaging session at the end of the longitudinal PET studies at 11.7 Tesla on a Bruker Biospec 117/16 USR scanner (Bruker Biospin, Ettlingen, Germany) using a volumetric and a surface coil for radiofrequency (RF) transmission and reception, respectively, and interfaced to an Advance III console, operated with Bruker’s software ParaVision 6.1. Anesthesia was induced with 4% isoflurane and kept at 1.5–2.0% during scanning, carried in 1.5 L/min mixture of N_2_/O_2_ (70/30). Animals were placed on an MRI-compatible holder and maintained at 37 ± 1 °C using a circulating water heating pad. Imaging protocol included the acquisition of: 1) 3 orthogonal T_1_W sets of images used as scout images for subsequent positioning of imaging planes. 2) A set of high resolution T_2_-weighted images achieved with a TurboRARE image sequence using a RARE factor of 8, repetition/effective echo time (TR/TE_eff_) = 4938/40 ms, a flip angle of 90º, a number of averages (Nav) = 8, a field of view (FOV) = 12.75 mm × 12.75 mm, a matrix size of 170 × 170 points (in-plane resolution of 75 µm), and 24 consecutive slices of 500 µm thickness (covering the whole brain in a 12 mm field of view along z axis). 3) a set of Diffusion Tensor Imaging (DTI) performed using a spin-echo based diffusion weighted imaging sequence (SE-DWI), using the following parameters: repetition time/echo time (TR/TE) = 1410/20 ms; b = 1000 mm^-2^s (δ = 4 ms and Δ = 11 ms), 40 gradient directions + 5 b0 images, Number of averages (Nav) = 1. Full brain was covered using a field of view FOV = 12.8 × 9.6 mm^2^, with a matrix size = 128 × 96 points (in-plane resolution = 100 µm), and 24 consecutive slices of 500 µm thickness. For image processing, first voxel dimension of all images was multiplied by 10 to improve the processing performance of FSL software library [FMRIB (Oxford Centre for Functional MRI of the Brain); <http://www.fmrib.ox.ac.uk/fsl>] [[5](#_ENREF_5),[6](#_ENREF_6)]. Additionally, diffusion images were denoised using local PCA-based algorithm [[7](#_ENREF_7)] implemented in Dipy library [[8](#_ENREF_8)] for python.

Next, all MR images were placed in the same space as a down-sampled in-house template. High resolution T_2_W images were used for achieving optimal brain extraction and registration to the template. T_2_-weighted images were skull stripped and bias corrected using FSL. Afterwards, registration to the in-house template was performed. Masks and transformation matrixes obtained for the T_2_W images were subsequently applied to each of the MR image modalities. Simultaneously, the publicly available AMBMC (Australian Mouse Brain Mapping Consortium, http://www.imaging.org.au/AMBMC/) mouse atlas was registered to the template for automatic regions of interest (ROIs) selection on images.

A series of regions of interest (ROIs) were selected and extracted from the anatomical atlas, covering both grey matter and white matter regions including: the cerebellar peduncles (CP), the hippocampus (HIP), the thalamus (THA), the caudate putamen (CPu), the motor cortex (MC), the cortex (CTX), including somatosensory, motor and auditory cortex. The CC was separated in the medial corpus callosum (med-CC) and the lateral corpus callosum (lat-CC), for image analysis.

Additionally, regional brain volumes (V) of grey matter areas were quantified by registration of the images to the AMBMC mouse brain atlas with 12 degrees of freedom.

*Ex vivo* studies

Immunohistochemistry and Thioflavin staining were carried out in 8-month-old brains in both 5xFAD and WT mice. Brains were removed, frozen and cut in 5-μm-thick sections in a cryostat. Sections were fixed in 4% paraformaldehyde during 15 min, washed with phosphate-buffered saline (PBS) and incubated 5 min in NH_4_Cl, following by two PBS rinse and methanol-acetone (1:1) permeabilization during 5 min at -20 ºC. After PBS washing, samples were saturated with a solution of bovine serum albumin (BSA) 5%/Tween 0.5% in PBS during 15 min at room temperature and incubated during 2 h at room temperature with primary antibodies BSA (5%)/Tween (0.5%) in PBS. Different brain sections containing cerebral cortex (CTX), hippocampus (HIP), thalamus (THA) and cerebellum (CB) were stained separately for Iba1 with guinea pig anti-rat Iba 1 (1:250; Synaptic Systens, Goettingen, Germany), GFAP with chicken anti-rat GFAP (1:500; AbCam, Cambridge, UK), TSPO (1:300; AbCam, Cambridge, UK) and xCT with rabbit anti-rat xCT (1/100; AbCam, Cambridge, UK) to evaluate the cellular expression of both TSPO and xCT in microglia/macrophages and astrocytes. Finally, brains were stained for 4-NHE with mouse anti-4NHE (1:250; AbCam, Cambridge, UK) to evaluate the formation of 4 hydroxynonenal as result of lipid peroxidation. Sections were washed (3 × 10 min) in PBS and incubated for 1 h at room temperature with secondary antibodies Alexa Fluor 594 donkey anti-rabbit IgG (Invitrogen Molecular Probes, Life Technologies, Madrid, Spain, 1:1000), Alexa Fluor 647 goat anti-chicken IgG (Invitrogen Molecular Probes, Life Technologies, Madrid, Spain, 1:1000) and Alexa Fluor 647 goat anti-guinea pig IgG (Invitrogen Molecular Probes, Life Technologies, Madrid, Spain, 1:500) in BSA 5%/Tween 0.5% in PBS, washed again (3 × 10 min) in PBS. Subsequently, sections were incubated with 0.01% Thioflavin S in 70% ethanol, diluted 1:10 in 0.1 M PBS for 10 min and washed with 0.1 M PBS. Finally, sections were mounted with a prolong antifade kit without DAPI in slices ((Invitrogen Molecular Probes Life Technologies, Madrid). Images were acquired with the Pannoramic MIDI II automated digital slide scanner (3DHistech Ltd., Hungary).

***Statistical analysis***

PET results were analysed using two-way analysis of variance ANOVA. Differences between groups (5xFAD vs WT) at each time point and differences between time points within each group were determined using Sidak’s multiple comparisons test. For MRI results, the statistical significance of the difference between groups was determined by a two-way ANOVA, followed by an unpaired t-test. Differences were concluded significant for P values < 0. 05: P < 0.05, *; P < 0.01, **, P < 0.001, ***; and P < 0.0001, ****. Statistical tests were performed in GraphPad Prism 7.03 (GraphPad Software, CA, USA).


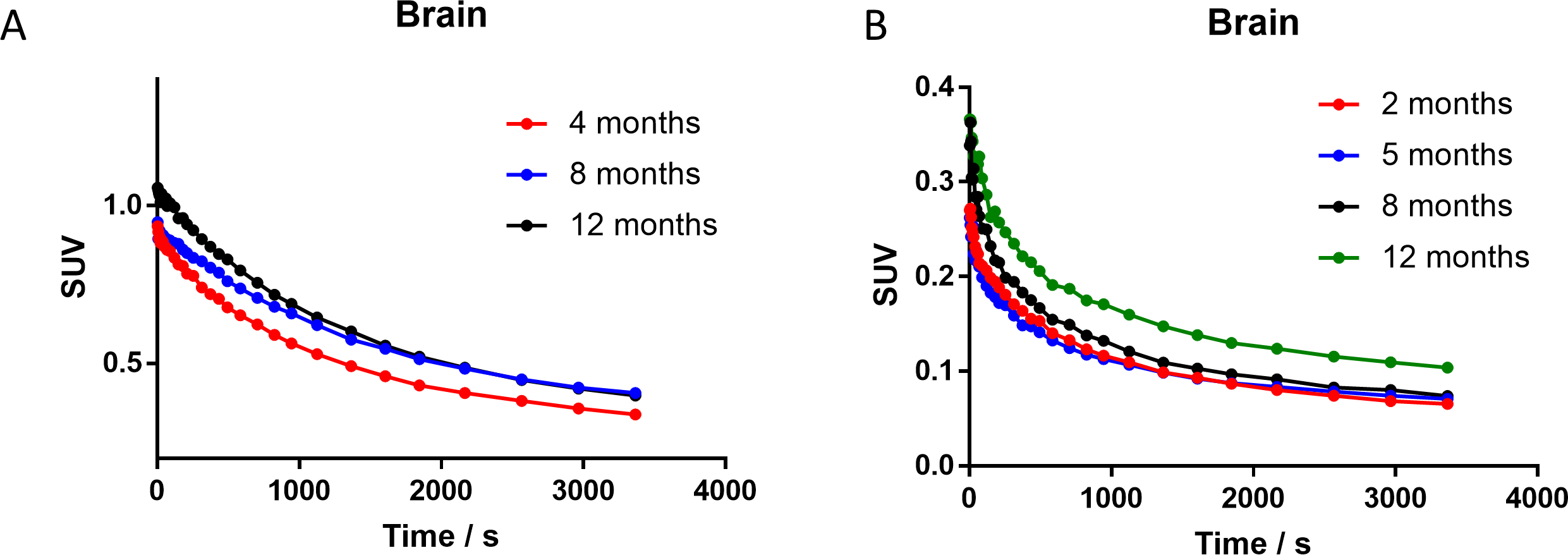


Figure S1. Time activity curves in the whole brain, expressed as standard uptake value (SUV) obtained after administration of [^18^F]DPA-714 (A) and [^18^F]FSPG (B) to WT mice at different ages.

**
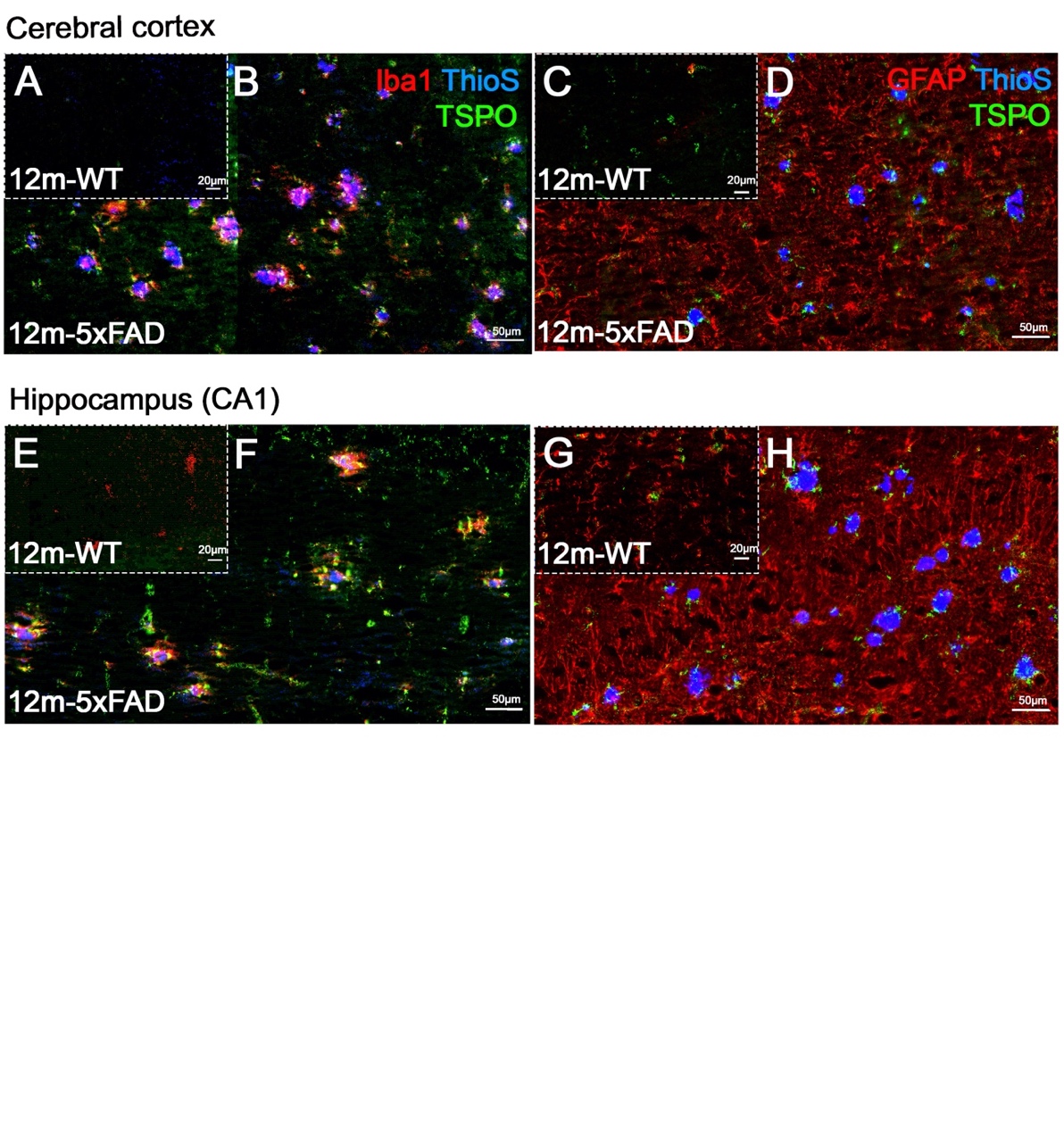
**

**Figure S2.** Staining of Iba1 or GFAP (red), Thioflavin S (blue) and TSPO (green) in 12 month-old brains of WT (A, C, E and G) and 5xFAD mice (B, D, F and H) shown as merged channels.

**
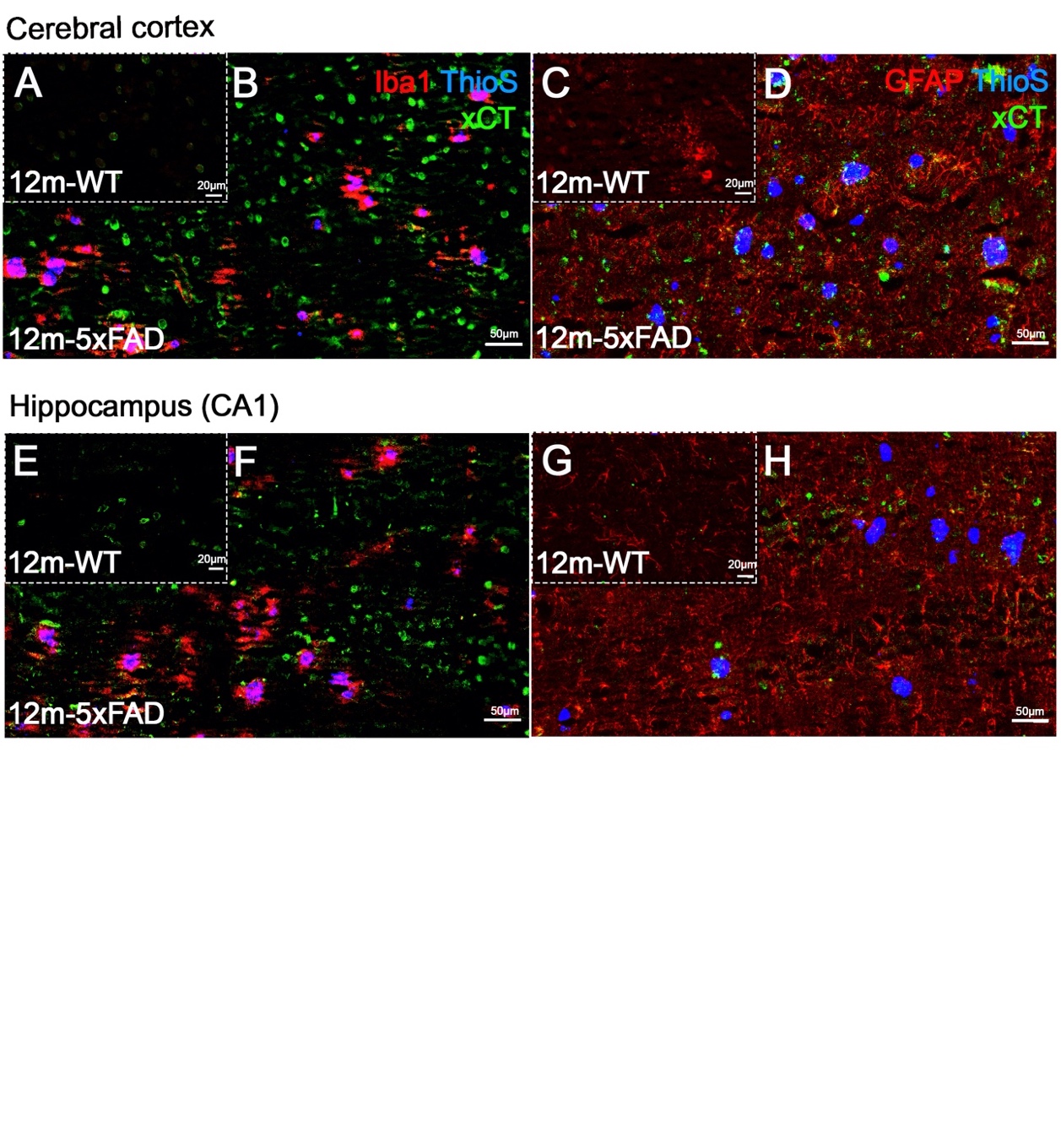
**

**Figure S3.** Staining of Iba1 or GFAP (red), Thioflavin S (blue) and xCT (green) in 12 month-old brains of WT (A, C, E and G) and 5xFAD mice (B, D, F and H) shown as merged channels.

**
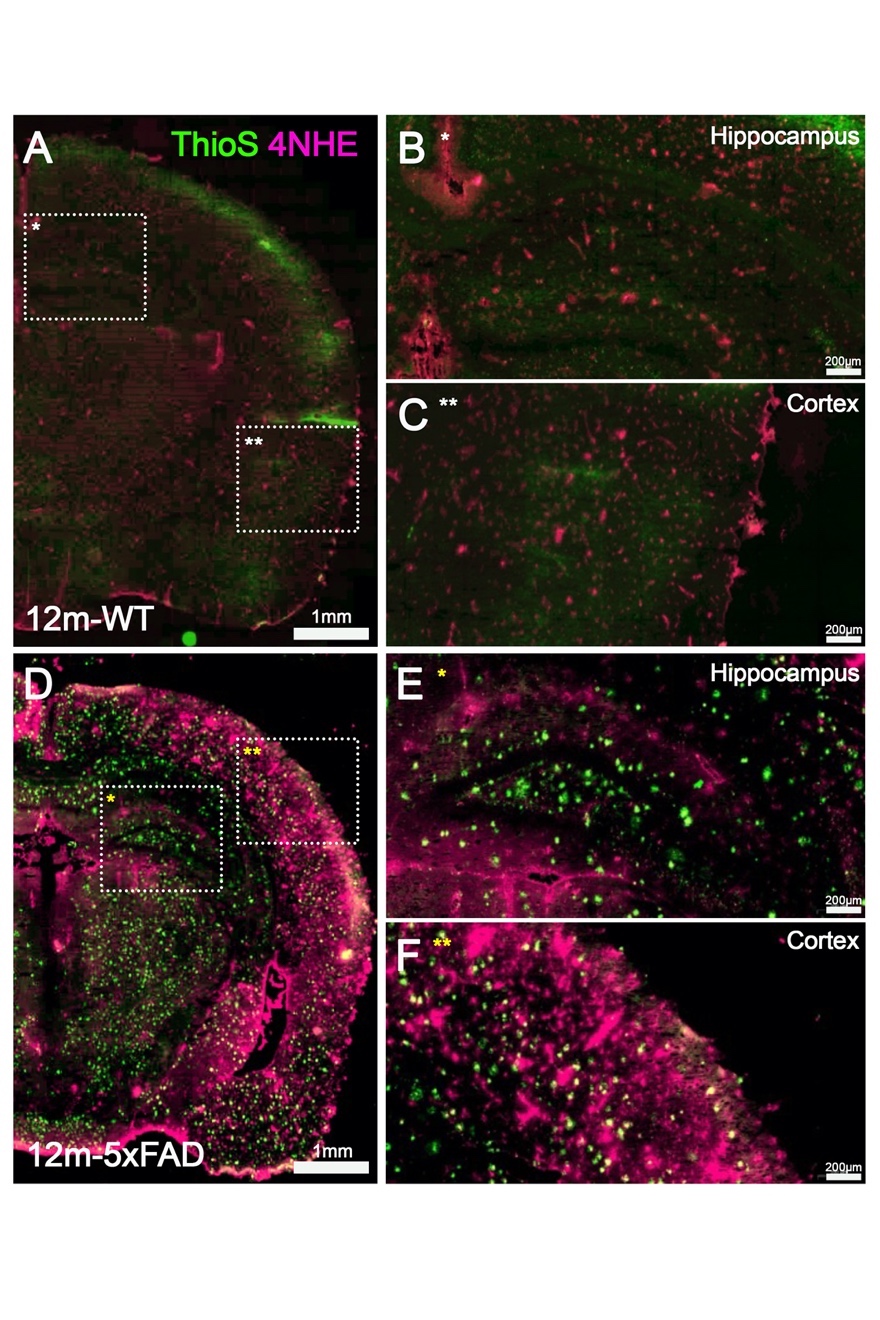
­­**

**Figure S4.** Staining of 4NHE (pink) and Thioflavin S (blue) in 12-month-old brains of WT (A-C) and 5xFAD mice (D-F) shown as merged channels.

**REFERENCES**

**1.** Pulagam KR, Colas L, Padro D, et al. Evaluation of the novel TSPO radiotracer [(18)F] VUIIS1008 in a preclinical model of cerebral ischemia in rats. EJNMMI Res*.* 2017;7:93.

**2.** Domercq M, Szczupak B, Gejo J, et al. PET Imaging with [(18)F]FSPG Evidences the Role of System xc(-) on Brain Inflammation Following Cerebral Ischemia in Rats. Theranostics*.* 2016;6:1753-1767.

**3.** Takkinen JS, Lopez-Picon FR, Al Majidi R, et al. Brain energy metabolism and neuroinflammation in ageing APP/PS1-21 mice using longitudinal (18)F-FDG and (18)F-DPA-714 PET imaging. J Cereb Blood Flow Metab*.* 2017;37:2870-2882.

**4.** Rejc L, Gomez-Vallejo V, Joya A, et al. Longitudinal evaluation of a novel BChE PET tracer as an early in vivo biomarker in the brain of a mouse model for Alzheimer disease. Theranostics*.* 2021;11:6542-6559.

**5.** Jenkinson M, Bannister P, Brady M, Smith S. Improved optimization for the robust and accurate linear registration and motion correction of brain images. Neuroimage*.* 2002;17:825-841.

**6.** Smith SM. Fast robust automated brain extraction. Hum Brain Mapp*.* 2002;17:143-155.

**7.** Manjón JV, Coupé P, Concha L, Buades A, Collins DL, Robles M. Diffusion Weighted Image Denoising Using Overcomplete Local PCA. PLoS One*.* 2013;8:e73021.

**8.** Garyfallidis E, Brett M, Amirbekian B, et al. Dipy, a library for the analysis of diffusion MRI data. Frontiers in Neuroinformatics*.* 2014;8.
